# Post-prandial tracer studies of protein and amino acid utilisation: what can they tell us about human amino acid and protein requirements?

**DOI:** 10.1017/S0007114524000734

**Published:** 2024-06-28

**Authors:** D. Joe Millward

**Affiliations:** Department of Nutritional Sciences, School of Biosciences & Medicine, Faculty of Health and Medical Sciences, University of Surrey, Guildford, UK

**Keywords:** Nitrogen balance, Tracer kinetic studies, Indicator oxidation, Adaptive oxidation

## Abstract

Nitrogen balance (NB), the principal methodology used to derive recommendations for human protein and amino acid requirements, has been widely criticised, and calls for increased protein and amino acid requirement recommendations have been made, often on the basis of post-prandial amino acid tracer kinetic studies of muscle protein synthesis, or of amino acid oxidation. This narrative review considers our knowledge of the homeostatic regulation of the FFM throughout the diurnal cycle of feeding and fasting and what can and has been learnt from post-prandial amino acid tracer studies, about amino acid and protein requirements. Within the FFM, muscle mass in well fed weight-stable adults with healthy lifestyles appears fixed at a phenotypic level within a wide range of habitual protein intakes. However homoeostatic regulation occurs in response to variation in habitual protein intake, with adaptive changes in amino acid oxidation which influence the magnitude of diurnal losses and gains of body protein. Post-prandial indicator amino acid oxidation (IAAO) studies have been introduced as an alternative to NB and to the logistically complex 24 h [^13^C-1] amino acid balance studies, for assessment of protein and amino acid requirements. However, a detailed examination of IAAO studies shows both a lack of concern for homeostatic regulation of amino acid oxidation and  major flaws in their design and analytical interpretation, which seriously constrain their ability to provide reliable values. New ideas and a much more critical approach to existing work is needed if real progress is to be made in the area.

## Graphical abstract

**Figure:**
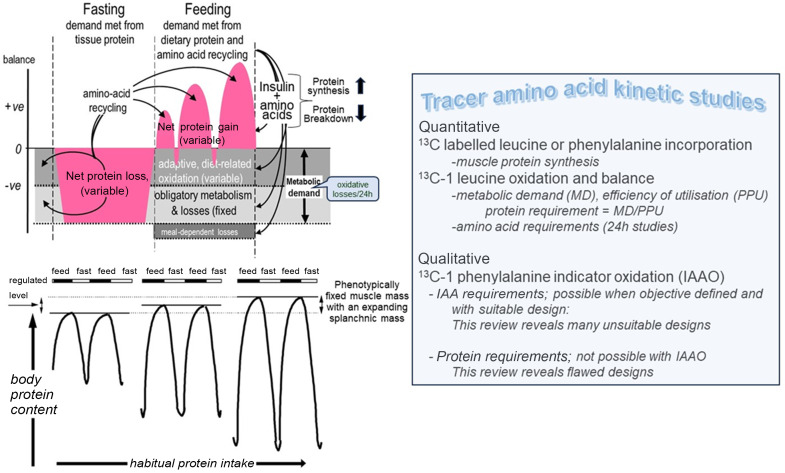

Recommendations for human protein requirements have always been subject to controversy and lack of consensus, and this remains the case today. Since the middle of the last century FAO and WHO have periodically published recommendations, the most recent being the FAO/WHO/UNU report of human protein and amino acid requirements published in 2007^(1)^, see Millward 2012^(2)^ and national and regional bodies (USDA, EFSA), usually follow these guidelines in their own deliberations, for example, IOM 2005^(3)^. The metabolic and physiological perspective of the 2007 report was identifying dietary protein and amino acid intakes which allowed the maintenance of the lean body mass in healthy well-nourished adult men and women of all ages and in population groups with special needs, namely infants and children during normal and catch-up growth during nutritional rehabilitation, in pregnant and lactating women, and during infection. In its review of the methods available for the determination of protein and amino acid requirements, the WHO 2007 report concluded that the estimation of human requirements for protein and amino acids remained an inherently difficult problem and that the only available method for estimating the requirement for total protein is as nitrogen measured by nitrogen balance (NB). Thus, NB studies in adults were evaluated through a meta-analysis allowing estimation of the requirements for adult men and women, with a factorial method used to derive values for children and pregnant women. It also concluded that no method is entirely reliable for determining the dietary requirement for essential amino acids (EAA). Thus, the recommended intakes of EAA were the mean estimates of values for each EAA derived by a variety of methods.

Since the 2007 report was published, a comprehensive set of NB studies in young and elderly adults was reported by Campbell et al. 2008^(4)^, which in the confirmation of the 2007 report’s adult safe level of protein intake of 0·83 g/kg/d identified a safe allowance of 0·85 ± 0·21 g/kg with no change with age. Although such a safe allowance is easy to achieve, being well below the median adult protein intakes of most developed societies such as the UK, for example, 1·24 g/kg per d, 13·7 % food energy in the UK elderly^(5)^, or 1·17 g/kg/d, 15·5 % energy in Dutch physically active elderly^(6)^, many have argued that the safe intake is too low^(7,8)^, that ‘Most adults benefit from protein intakes above the minimum RDA’^(9)^ and that the NB approach is unsatisfactory (e.g. Humayun et al. 2007^(10)^), for a variety of reasons. The context of this dissatisfaction has almost invariably been post-prandial studies of amino acid or protein metabolism of one sort or another. Some of these studies involve measurement of muscle protein synthesis (MPS) in response to amino acid or protein intake, for example, Paddon-Jones et al. 2015^(8)^ with some also investigating whole-body protein kinetics at the same time Kim et al. 2016^(11)^. Whilst such studies have not involved identification of a protein requirement *per se,* they have been the basis for recommendations of the amounts and timing of protein intakes in meals thought to maximise MPS as a proxy for the maintenance of the muscle and lean body mass^(12,13)^, recommendations which are now quoted as deriving from experts^(14)^. Others have developed studies of amino acid oxidation in response to varying amino acid or protein intake which do purport to inform directly on amino acid and protein requirements^(10,15,16)^, and these studies will be specifically evaluated here.

One difficulty in evaluating any of this work in the context of overall amino acid and/or protein requirements is that much of the work has not occurred within a generally agreed metabolic framework within which regulation of the lean body mass occurs. Thus, in the context of studies on the stimulation of MPS and the maximal anabolic response to dietary protein in a meal, Wolfe and colleagues^(17)^, although recognising that there must be some level of protein intake beyond which no further gains in net balance can occur, conclude that ‘Consistent with the theoretical calculations, experimental data show that the net gain in body protein in response to dietary protein intake is linear over a large range of protein intake’ and that ‘it is not likely that there is a practical limit to the maximal anabolic response to a single meal, and the most efﬁcient way in which to maximise the total anabolic response over a 24-h period is to increase dietary protein at breakfast and lunch without reducing protein intake with dinner’. This does not seem entirely consistent with their statements in the introduction to their paper recognising an upper limit to net gain of body protein after a meal. Their arguments about no practical limit to the maximal anabolic response to a meal are made on the basis that the feeding response not only involves an increase in MPS as measured in muscle biopsy studies but also includes the suppression of protein breakdown. Whilst Wolfe and colleagues are quite right to point out the importance of suppression of protein breakdown as part of the anabolic response to feeding, this is hardly a new observation^(18,19)^. Their comments also include the implicit assumption that both muscle and the lean body mass are a simple reflection of protein intake and are not specifically regulated.

As for the post-prandial oxidation studies, it is argued that ‘Determination of amino acid requirements involves feeding of graded levels of the test amino acid to the subject and looking for a clearly deﬁnable change in a relevant biological parameter’ and ‘Fundamentally, all of the methods used are a surrogate for measuring protein synthesis, which is hard to measure directly’^(15)^. Clearly, such studies are most important given that amino acid requirement values are necessary to devise a scoring pattern to judge the protein quality of dietary protein^(20–22)^. Whilst these latter authors have on different occasions discussed issues relating to the design of indicator amino acid oxidation (IAAO) studies which might influence their response curves, most recently reviewing the need for prior adaptation^(23)^, they do not discuss the wider metabolic context of their studies, especially the differences as identified by David Baker^(24)^ between requirements for growth in young animals and for maintenance in human adults and slow-growing children.

The issue considered here is that assuming a definition of the protein requirement as defined above by WHO/FAO, the maintenance of the lean body mass and the provision for growth in children and special groups, what do we know about the impact of varying protein intakes on homoeostatic regulation of the fat-free mass (FFM) and what can post-prandial studies tell us about amino acid and protein requirements? These are important questions for several reasons. First in the case of amino acids although there is a large literature about the important metabolic roles for individual amino acids^(25)^, especially in relation to clinical nutrition and cancer treatment^(26)^, and whilst this issue was discussed to a limited extent at the 2013 FAO consultation on protein quality evaluation^(22)^, suitable methodologies relating to quantifying the roles of amino acids in specific pathways which could result in identification of dietary guidelines for their use in clinical nutrition have yet to be devised. Thus, the major debate on the requirements of amino acids remains that of agreeing values for the nine EAA used to devise scoring patterns for protein quality evaluation, and this has been the main focus of post-prandial studies of amino acid requirements. Second as far as values for the protein requirement, much of the recent concern has focused on the issue of optimal protein requirements with the implicit assumption that such values will be higher than current recommendations for a variety of reasons^(13,27)^. The authors review of the literature in 1999^(28)^ and in relation to the amelioration of sarcopenia in the elderly in 2012^(5)^ was unable to find a convincing case for benefit from higher protein intakes. Furthermore, although the protein report by WHO/FAO made clear recommendations about the additional protein and amino acid requirements in various disease states and infections, it was unable to identify any clear quantifiable information about the relationship between protein intake and health and was, as a result, not able to identify *optimal* protein intakes with any certainty. However, there is a growing literature which would suggest caution in any proposal that recommended intakes of protein should be increased. For example genome-wide association studies of relative intakes from the macronutrients fat, protein, carbohydrates and sugar in over 235 000 individuals of European ancestries have identified phenotypes in which their relative protein intake exhibits a relationship with poor health, including positive genetic associations with obesity, type 2 diabetes and heart disease^(29)^. The authors argue that their findings are consistent with a literature that is supportive of such a relationship. One such recent report comes from Mittendorfer and Razani^(30)^ It is important therefore that studies advocating the need for higher protein intakes are examined carefully.

## Protein intakes and the fat-free mass

It appears that healthy body weights of adults can be maintained on a wide range of diets with protein intakes which are quite variable according to their dietary choices and lifestyles^(31)^, with meat consumption associated with much higher protein intakes than in vegetarian communities. Whether the higher protein intakes in meat eaters are associated with increased FFM is difficult to identify since meat eaters generally have higher BMI values than vegetarians, and whilst weight gain is associated with increased FFM^(32)^, measurements of the FFM are seldom measured in epidemiological studies of diet and health. In the EPIC-Oxford study of health conscious UK adults^(33)^, in which lacto-ovo-vegetarians and vegans consumed diets with protein comprising 14 and 13·1 % energy compared with 17·2 % for meat eaters^(34)^, the selection of health conscious subjects resulted in only minor differences in BMI between their dietary groups (24·3 kg/m^2^ meat eaters and 22·3 kg/m^2^ vegans), although these differences were associated with increased measured blood pressure and self-reported hypertension^(33)^ which reflected BMI. This suggests the differences in BMI reflected adiposity. In fact in the absence of resistance exercise or weight gain, there is little evidence that increased dietary protein intakes influence muscle mass or function, although few properly conducted trials have been reported or objectively discussed. For example, an RCT of protein supplementation at 31 g protein/d in physically active older Dutch adults selected on the basis of protein intakes ≤ 1·0 g/kg/d reported no change in lean body mass or any measure of muscle strength or physical performance^(35)^. However, because of a slightly (unexplored) greater weight loss in the supplemented group, lean body mass as a % of body weight increased marginally (*P* = 0·046), and this was the main finding of the trial reported in the authors abstract. A recent systematic review and meta-analysis of RCTs of the effects of increased dietary protein intakes on lean body mass gain, skeletal muscle strength and physical function in healthy adult subjects identified only six studies which did not involve resistance exercise training. Of these, there were no significant effects on lean body mass (6/6), bench press strength (1/1), lower-body strength (4/4), or handgrip strength and functional or physical test performance (4/4)^(36)^. On this basis, it appears that the phenotypic skeletal muscle mass in well-fed adults with healthy lifestyles and not engaging in specific resistance exercise does not vary as a function of their protein intake within a wide range of intakes.

## Post-prandial protein metabolism

The introduction of stable isotope studies, and especially GC-MS techniques, in the late 1970s, facilitated human studies of the response of whole body^(37)^ and muscle protein turnover^(38)^ to feeding. These early studies were largely exploratory demonstrating that protein turnover in humans *in vivo* could be measured and, as animal studies had shown^(18)^, was responsive to protein intake. Subsequently, we undertook more focused studies to investigate the response of whole-body protein turnover and homoeostasis in response to varying protein intake in two series of studies.

In the first series, the diurnal changes in both N balance and amino acid balance and turnover were measured with multiple-stable isotope tracers (l-[^13^C-1]leucine, l-[ring-^2^H_5_] phenylalanine, l-[ring-3, 5–^2^H_2_] tyrosine and l-[^15^N]glycine at the end of 2-week periods of adaptation to increasing protein intakes over a wide range, from low, 0·36 g/kg/d, to high, 2·31 g/kg/d, protein intakes. The tracer studies involved an 8-h fasting-feeding protocol initiated in the last 4 h of fasting and continuing during the first 4 h of feeding of hourly small meals to maintain a metabolic and isotopic steady state. On the basis of both 12-h N-balances, corrected for changes in size of the body urea pool, and [^13^C-1] leucine balances, a nutritionally sensitive diurnal cycle of increasing fasting N and protein losses and fed state gains with increasing protein intakes was observed^(39,40)^. The same protocol was also used to study the time course of the adaptation of the diurnal cycle of N homoeostasis over 9 and 14 d during a change in protein intake from a high, 1·8–1·9 g/kg/d, to a moderate, 0·77 g/kg/d, protein intake^(41)^. This showed that adaptation of amino acid oxidation to a change in protein intake was slow and incomplete over the short time period of our studies and involved some transient weight loss while the adaptation was occurring. These and other human studies, together with animal studies of the regulation of muscle growth^(42)^, resulted in the formulation of a ‘protein-stat’ mechanism for the regulation of growth and maintenance of the lean body mass explaining the interaction between dietary protein intake and whole body and muscle protein homoeostasis and growth^(43)^, which has recently been updated in the context of childhood growth and development^(44)^.

The key feature of the protein-stat concept is the interaction between bone length growth and appendicular muscle mass, whereby a phenotypical specific capacity for muscle growth is established through mechanotransduction mechanisms. Thus, the diurnal cycle of post-absorptive losses and post-prandial gains will occur to maintain muscle mass at the maximum, phenotypically controlled level, at which further protein deposition in muscle is limited by a ‘bag full’ signal^(42–44)^. Any additional protein intake above the habitual level can be deposited within the splanchnic tissues to a limited extent and will then be diverted to lipogenesis and adipose tissue expansion. Evidence for a limit to post-prandial protein deposition in muscle was first indicated by Rennie and Wolfe^(45)^ and subsequently by Atherton’s group^(46–48)^. Thus, contrary to Wolfe’s statement that ‘it is not likely that there is a practical limit to the maximal anabolic response to a single meal’^(17)^, there does seem to be a limit at least in terms of the anabolic response of MPS. For those with habitual high-protein intakes with increased post-absorptive losses, there will be an increased capacity for post-prandial gain, but there is little to suggest the overall size of the muscle mass is increased.

**Fig. 1.**
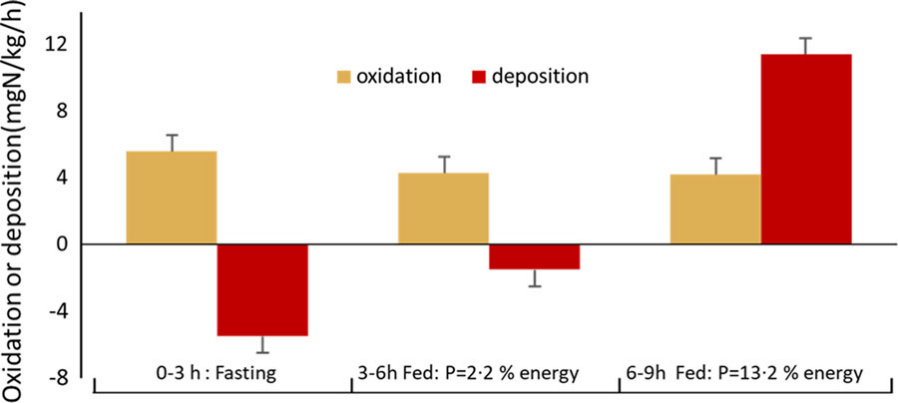
Partition of dietary protein intake between oxidation and tissue protein deposition with meals of low and high protein in human adults^(51)^. Meal protein utilisation was measured during 9 h (3 × 3 h), [^13^C-1] leucine balance studies in subjects studied sequentially in the post-absorptive state following by repeated small meal, milk-based intakes of low-protein and then high-protein meals, consumed at an hourly rate of one-twelfth of daily energy requirements. Balance in terms of nitrogen was calculated from leucine oxidation and balance taking into account the relative leucine contents of milk and tissue protein. The low-protein meals reduced both the post-absorptive losses of tissue protein and, to a lesser extent amino acid oxidation, while the high-protein meals induced tissue protein gain and no further change in amino acid losses. In response to the high-protein meal, although the efficiency of protein utilisation during the LP-HP transition was 100 % based on the changes in N deposition/N intake, of the total N intake on the HP diet 73 % was deposited and 27 % oxidised.

The second series of studies evolved out of a stable isotope protocol designed to study the mechanisms of the regulation of post-prandial protein utilisation (PPU)^(49)^ and to quantify its efficiency,^(50–52)^. These studies, the first to establish the efficiency of PPU (the equivalent of the classical term NPU, net protein utilisation), were important, since it had been suggested in the 1985 FAO/WHO/UNU protein and energy requirements report that the elderly needed more protein because of an age-related decline in protein utilisation, although no unequivocal evidence had been cited to support this. Also it was noted in the 2007 report^(1)^ that the efficiency of protein utilisation from high-quality dietary sources indicated in N-balance studies was usually very low, often ≤ 50 %, which was biologically implausible. The protocol initially employed^(49)^ involved a 9-h primed continuous infusion of [^13^C-1] leucine involving 3 × 3 h periods, initially in the post-absorptive state followed by the feeding of frequent small meals to enable a metabolic and isotopic steady state equivalent to daily intakes of a low-protein diet (3 % energy) and then isoenergetic higher protein meals (15 % energy). Leucine oxidation and balance were determined from plasma [^13^C-1]-α-ketoisocaproate enrichment and expired ^13^CO_2_ excretion measured during the third hour of each 3-h period. The protein intake during the third phase was similar to the habitual intake estimated in the subjects from 24-h urinary N excretion. Because the two consecutive feeding periods were isoenergetic, insulin levels were maintained at a constant level and the changes in leucine balance during these feeding phases could be assumed to reflect the responses to amino acids from the protein fed in period 3, while the balance change between period 1 and 3 reflected the overall response to the meal (energy + protein). The feeding mechanism identified in these studies was the combination of an insulin-mediated, protein-conserving influence of dietary energy that inhibited whole-body protein degradation, lowered amino acid levels, and reduced oxidation, and an amino acid-mediated augmentation of the inhibition of degradation, a stimulation of synthesis, and an increase in oxidation when leucine dietary supply exceeds the capacity for its net deposition^(19,49)^. Typical responses to frequent small meals of low and high protein are shown in Fig. 1 ^(51)^. Most importantly, PPU could be better defined in terms of the meal-related losses, that is, as changes in leucine oxidation, low protein to high protein, as a proportion of the intake, corrected for the difference between leucine content of the meal and tissue protein^(52)^. The efficiency of protein utilisation, the PPU_nitrogen_ value, was what would be expected, high for milk, close to unity (100 %) and lower for wheat^(51)^. Of the total N intake on the HP diet, 73 % was deposited and 27 % oxidised.

### Adaptive metabolic demands and the protein requirement

**Fig. 2.**
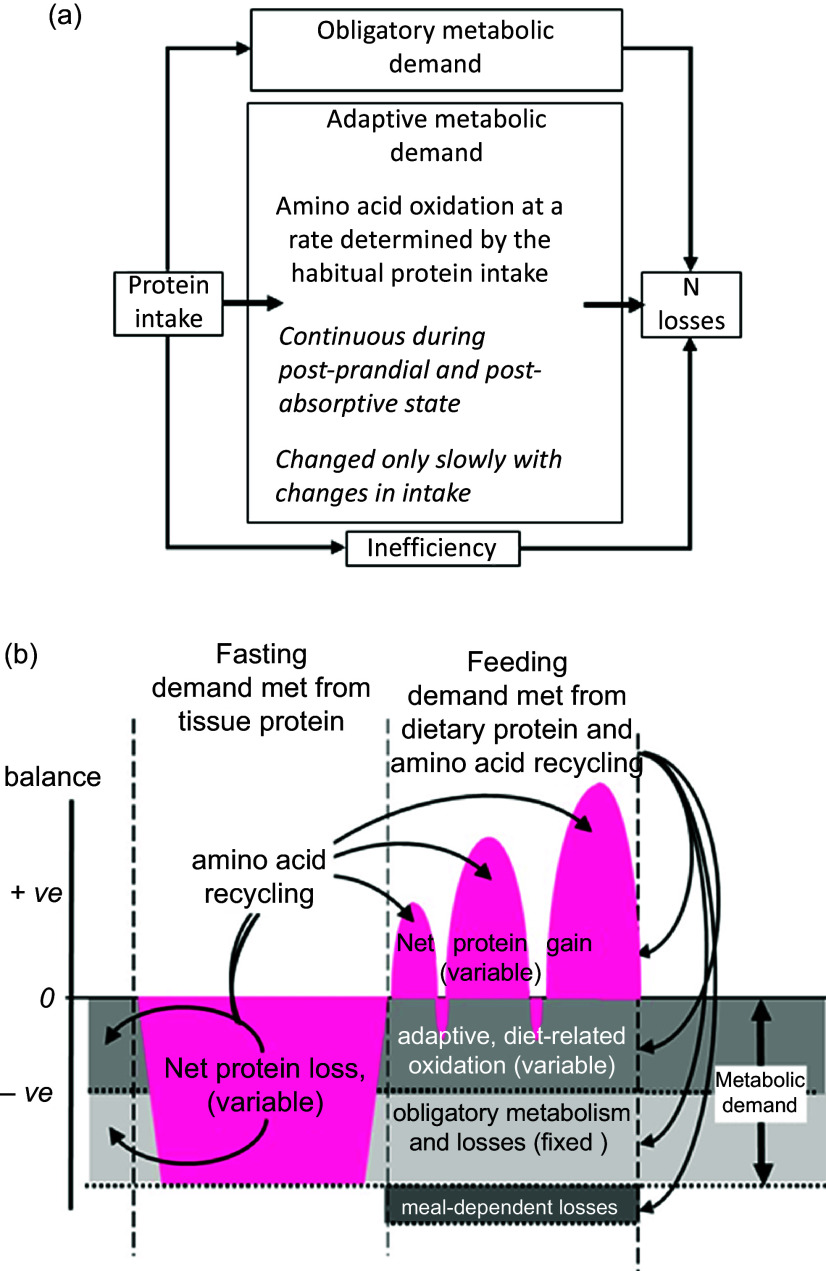
Adaptive metabolic demands model of the protein requirements. The fate of dietary protein at overall amino acid balance, that is, at maintenance. (a) Amino acids from dietary protein are consumed by both the obligatory metabolic demand in which amino acids are consumed for essential metabolic purposes and by adaptive oxidative losses resulting in each case in N excretion. (b) The detailed fate of amino acids during the diurnal cycle. Net losses of tissue protein during fasting occur to meet the overall obligatory and adaptive metabolic demand with net gains of tissue protein during feeding when dietary amino acids meet the needs of both deposition and the obligatory and adaptive metabolic demands with any excess amino acids oxidised. The amplitude of gains and losses of tissue protein varies according to changes in adaptive oxidation in response to variation in habitual protein intake. Some recycling of EAA occurs for those EAA with larger intracellular pool sizes like lysine and threonine. The overall metabolic demand at any level of habitual protein intake is measurable as post-absorptive losses scaled to 24 h; see Millward 2003^(53)^.

The results of these two sets of studies of diurnal cycling and PPU enabled us to formulate a new model for the protein requirement based on the concept of an adaptive metabolic demand (MD)^(53)^. The model (Fig. 2(a)) identiﬁes MD for amino acids for maintenance as comprising a small ﬁxed component comprising those processes which irreversibly consume amino acids for various purposes and which contribute to the obligatory nitrogen losses, and a variable adaptive component driven by oxidative losses of the EAA. One possible metabolic explanation of this relates to the homoeostatic mechanisms which maintain the low tissue concentrations of the potentially toxic branched chain, aromatic and *S*-containing amino acids^(54,55)^. Thus, during the usual relatively slow growth in childhood, and at weight maintenance in adults, the supply of these amino acids from food protein will usually be in excess of minimal needs so that they must be rapidly disposed to avoid excessive post-prandial increases in their tissue concentrations. This requires that the capacity of the pathways of oxidative catabolism of these particular amino acids adapts to match the habitual protein intakes^(56,57)^. Although these pathways are to some extent regulated by feeding and fasting, this regulation is only partial so that amino acid oxidation continues to occur after dietary protein is disposed of, continuing in the post-absorptive state with net catabolism of tissue protein. This means that in practice, adaptive oxidation is relatively insensitive to acute food or protein intake but does change slowly with a sustained change in intake, enabling N equilibrium to be achieved eventually within the range of intakes to which adaptation can occur. The model accounts for the apparent low efﬁciency of utilisation of all protein in N balance studies (see Millward 2003^(53)^ for further discussion).

As shown in Fig. 2(b) in the fasted state, tissue protein is mobilised to provide amino acids for both the fixed, obligatory and variable (adaptive) components of the MD. With feeding, dietary amino acids are utilised to replace tissue protein mobilised in the post-absorptive state and to provide for the obligatory and adaptive MD. As previously demonstrated^(51)^ and discussed^(20,52)^ for some amino acids, recycling may occur, namely those liberated in the fasted state which are not completely oxidised. However, the extent of this is difficult to quantify but will vary according to the kinetics and sensitivity to changes in pool sizes of the catabolic pathways of each amino acid but is more likely with lysine^(51)^ Recycling may also vary with the habitual level of protein intakes which governs the amplitude of fed state gains and fasting losses of tissue proteins^(20)^. The amino acid pattern of the demand which is for both protein deposition and for the obligatory and adaptive components of oxidative losses is difficult to predict^(20,52)^. Protein deposition requires amino acids which match the composition of tissue protein, although any recycling as shown will to some extent reduce the dietary demand for those amino acids involved, as will the amplitude of the gains and losses. Subjects with higher habitual protein intakes will display an increased amplitude of tissue gains and losses and although the extent of recycling may be increased, the EAA requirement pattern is likely to increasingly reflect that of tissue protein as with rapidly growing animals when the maintenance component will be a proportionally smaller component.

Figure 2(a) shows that any excess amino acid intake is oxidised (shown as meal-dependent losses), and it is the extent of this component that determines the efficiency of dietary protein utilisation. Thus, more realistic efﬁciency values can be measured within an experimental framework that takes account of the adaptive MD. In contrast to the current requirements model, for fully adapted individuals, risk of deﬁciency (i.e. negative N balance after complete adaptation) will only start to increase when habitual intakes fall below the range of the true minimum requirement. This value is currently unknown but may be at the lower end of the reported distribution of requirements as indicated by N balance studies, that is, between 0·4 and 0·5 g/kg per d^(58)^, or at intakes at the lower end of the Acceptable Macro-nutrient Distribution Range or AMDR for protein: 10–35 % of daily energy intakes^(3)^. At intakes greater than this within the AMDR, the additional MD vary directly with the intake so that deﬁciency is only likely as a short-term response to a change to a lower intake within the adaptive range. In practice, few natural diets which provide requirement levels of all other nutrients provide protein intakes as low as this.

On the basis of measured MD (post-absorptive losses scaled to 24 h) in subjects on their habitual protein intakes or after 2 weeks adaptation) and PPU (fractional efﬁciency of protein utilisation (utilisation/intake)) calculated from ΔN or leucine balance, an apparent protein requirement can be calculated as MD/PPU. For young and middle-aged men and women on habitual diets estimated at 1·16 ± 0·08 g/kg/d, apparent requirements were 0·80 ± 0·13 g/kg/d falling to somewhat lower values in the elderly because of significantly lower values for the MD^(50,59)^.

This adaptive MD model of the protein requirement raises difficult issues for nutritional recommendations as indicated above^(53)^ and has not to the authors knowledge been either specifically deployed by other investigators to assess human protein requirements. It has also attracted some controversy^(23,60–62)^, but to date it is arguably the only model which has been proposed which accounts for all the observed characteristics of post-prandial metabolism and NB studies.

## Post-prandial indicator amino acid oxidation

By far, the most extensive post-prandial studies of protein and amino acid metabolism are those involving IAAO, mainly published by Pencharz, Ball, Elango and their colleagues^(15,16,63)^, and this review will be limited to their evaluation. Of these, several studies were used to help define EAA requirements in the 2007 WHO report^(1)^ and in the US report from the Institute of Medicine^(3)^, although these studies were identified as involving a different paradigm to that of nitrogen or carbon balance. As discussed by the authors of IAAO^(64)^, IAAO is based on the concept that when an EAA is limiting for protein synthesis, the other amino acids will be oxidised (because they are present in relative excess), and this partition between retention and oxidation can be determined through the oxidation of a separate indicator amino acid in response to varying intakes of the test EAA. IAAO has been deployed to examine requirements for EAA, protein and, as discussed below, adaptive changes in post-exercise anabolism. Matsumoto et al.^(65)^ has published a scoping review of the evaluation of protein requirements using the IAAO method, identifying sixteen IAAO studies of which thirteen involve phenylalanine oxidation and meals of amino acids with fixed limiting intakes of phenylalanine and tyrosine, one involves phenylalanine oxidation and meals of protein with fixed unlimiting intakes of phenylalanine and tyrosine^(66)^ and two are quite different studies of leucine oxidation at varying protein intake levels. Of the studies involving a phenylalanine indicator, all derive from or are in collaboration with Paul Pencharz apart from those of Daniel Moore’s group. These various applications will be discussed separately here. IAAO has also been proposed by Elango, Ball and Pencharz as a suitable method to study dietary protein quality in terms of ‘Metabolic Availability’^(67)^ with several studies published, for example, Rafii et al. 2018^(68)^. Although these studies are by no means straightforward, they will not be examined here.

## Indicator amino acid oxidation studies of essential amino acid requirements: background to the methodology

**Fig. 3.**
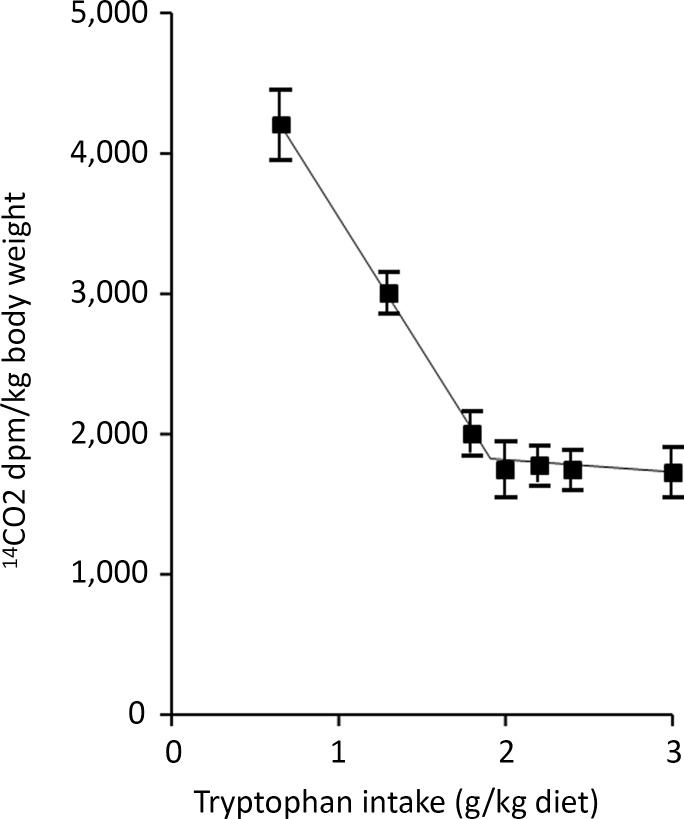
Application of the IAAO approach to measure amino acid requirements in piglets^(73)^. The influence of the dietary tryptophan intake on the oxidation of phenylalanine in piglets as assessed in post-prandial feeding studies. The animals were fed two sequential liquid meals over 4 h containing a mixture of skim milk (40 % protein) and amino acids (60 % protein) at 240 g/kg diet, an intake inducing maximum growth rates, containing variable amounts of tryptophan as indicated and with phenylalanine and tyrosine contents at concentrations found in skim milk. [^14^C]phenylalanine was added as the indicator amino acid, and ^14^CO_2_ was collected over 60 min in expired breath 2 h after the second meal. The data were analysed with a two-phase linear regression crossover model which allows a partition of the data points between the two separate linear regression lines with the intersection interpreted as the dietary requirement for tryptophan. The design concept is that the increasing tryptophan intake will improve the partition of the dietary amino acids between the demand for tissue protein deposition and amino acid oxidation, with oxidation falling to a minimum when the intake becomes balanced relative to the demand. This partition is measured as oxidation of the [^14^C-1] trace-labelled ‘indicator’ amino acid, phenylalanine in the diet which is assumed to reflect the overall partition of the dietary protein between oxidation and deposition. The ‘requirement’ tryptophan intake will be that which allows the amino acid mixture to match the demand. At this intake, higher tryptophan intakes will have no further influence on protein utilisation and consequent amino acid oxidation. Data redrawn from Ball and Bayley, 1984^(73)^.

Apart from reviews by Pencharz, Ball and their colleagues, no independent detailed analysis of IAAO studies of requirements for EAA has been published to the authors knowledge apart from a brief explanation in the 2007 WHO report^(1)^ and by Matthews^(69)^. IAAO studies, although presented as simple dose–response studies with a clear endpoint, are in fact quite complex, particularly in terms of the way they are designed and the data interpreted in human studies. The method originated from work by Henry Bayley on amino acid metabolism in young rapidly growing pigs^(70–72)^ which developed into the determination of amino acid requirements in the pig by means of the oxidation of an indicator amino acid^(73–76)^. The experimental approach involves assessing the requirement for an individual EAA to optimise the composition and hence the utilisation of meal protein, during feeding. Typical results are shown in Fig. 3, a study of the tryptophan requirement of piglets^(73)^. This involved acute post-prandial feeding studies with meals at a specific protein intake known to optimise piglet growth, comprising a mixture of actual protein (40 %) and free amino acids (60 %), within which the test amino acid content varied from a low level to excess compared with its usual content in milk protein. The oxidation of a trace-labelled indicator amino acid, usually 1-^14^C-labelled phenylalanine, added to the test meals acts as an ‘indicator’ for the oxidation of the meal amino acids in their partition between oxidation and net protein deposition. It is assumed that the test amino acid itself is utilised efficiently for protein deposition at all intakes until it is in excess, if that occurs, when it will accumulate and be oxidised, although this oxidation at excess intakes will not be observed as indicator oxidation. At the lowest test amino acid intakes, utilisation of the protein will be low and oxidation will be high which will be reflected in the partition of the indicator towards oxidation. Increasing test intakes will improve protein utilisation with decreasing oxidation until the overall meal amino acid profile is optimised, when this profile matches that of the demand. This is assumed to be the requirement intake. Any further increase in the test amino acid will not influence utilisation, so the indicator oxidation rate will remain low and constant. With these rapidly growing piglets, the change point in the 1-^14^C-labelled phenylalanine oxidation response to variation in the intakes of histidine^(74)^, the sulphur amino acids^(75)^, lysine and threonine^(76)^ and tryptophan^(73)^ was quite obvious and allowed fitting of a two-phase regression model allowing ‘an objective assessment of the change-over point, and its 95 % confidence limits for the corresponding dietary intake’^(74)^. Importantly, it was shown that the change point was the same when the trace-labelled phenylalanine oxidation was expressed as either recovered ^14^CO_2_ in breath or actual phenylalanine oxidation calculated from ^14^CO_2_ excretion adjusted for the specific radioactivity of liver-free phenylalanine. Thus, evaluation of the exact level of phenylalanine oxidation became less important than identifying a change point in the response^(73)^. In effect, the paradigm is the determination of the amount of intake of the test EAA that renders an amino acid mixture fully competent to meet the post-prandial demand, an intake which will be the growth requirement for the piglet.

Henry Bayley’s work on the amino acid requirements of the piglet was at a time when such work had been described by David Baker in his 1986 review of animal studies of essential nutrient requirements^(24)^. He stated that ‘Requirements for amino acids and most vitamins are generally best defined in growing animals by growth data in *ad libitum* feeding studies’. However, Bayley and Ball moved on from identifying growth responses to indicator oxidation responses as a more sensitive and convenient approach. Baker had also stated that ‘Obviously, problems are encountered in designing (mineral) requirement studies for adult animals and for both growing and adult humans. Since growth data are meaningless in adults…’. In fact, Malcolm Fuller devised dietary amino acid deletion studies to determine maintenance amino acid requirements of pigs, identifying a pattern of EAA requirement quite different to that of the EAA in tissue protein which determines the requirement pattern for growth^(77)^. In the tryptophan studies shown in Fig. 3, tryptophan requirements for nicotinic acid synthesis (i.e. part of the maintenance requirement for this amino acid) were removed from the study by feeding excess nicotinic acid^(73)^.

The IAAO approach to the measurement of the EAA growth requirements of piglets became the model for its application in human studies^(15)^, presumably on the basis that the anabolism during the post-prandial phase of the diurnal cycle of gains and losses during weight maintenance in human adults was equivalent to studying animal growth responses (although this has never been specifically discussed in any of the human IAAO studies, or in reviews of the methodology). There are however certain important differences. The meals fed usually comprise an amino acid mixture based on the amino acid composition of egg protein. Most importantly, the intakes of the indicator and in some cases other amino acids were fixed at levels considerably below that found in egg or other animal-source food proteins, or in tissue protein. The importance of the dietary concentration of the indicator had been examined in the pig studies to avoid both an underestimate of the test EAA by providing too little and a reduction of the sensitivity of the oxidation responses through excessive oxidation of the indicator, when in excess^(74)^. Studies of phenylalanine oxidation with increasing phenylalanine intake showed that the provision of more than 7 g of phenylalanine/kg diet (which contained 240 g/kg protein) resulted in an excess (increasing phenylalanine oxidation), indicating that this was the dietary requirement under the conditions used in the determination. In fact, given the phenylalanine concentration in tissue protein (discussed below), it can be calculated that 7 g/kg would have allowed the deposition of 75 % of the protein consumed by the piglets. As a result, subsequent studies of EAA requirements of the piglet with IAAO involved phenylalanine intakes of either 8·0 g/kg^(76,75)^ or10·7 g/kg^(73)^.

The level of the phenylalanine indicator fed in the human studies resulted from a study of phenylalanine flux, oxidation and conversion to tyrosine^(78)^ which reported phenylalanine oxidation studies which were interpreted as indicating that the phenylalanine requirement with excess tyrosine was 9·1 mg/kg/d. In fact on the basis of a tissue protein content of 44 mg phenylalanine/g protein, 9·1 mg/kg/d will allow the deposition of only 200 mg of tissue protein, a small fraction of the total amino acid mixture fed. All subsequent studies depended on this result to choose the concentration of the phenyalanine indicator. The value chosen (shown in Table 1 below) was described as 120 % of the requirement, but it was, in fact, much less than the requirement for efficient utilisation of the meal amino acids.

**Table 1.** Influence of design on outcome of IAAO studies of EAA requirements

1		2	3	4	5	6	7	8	9	10	11
Study reference		Test AA	Subjects	AA mixture intake: g/kg/d	Intake of indicator mg/kg/d	Max protein deposition from indicator %intake*	Test AA intake range mg/kg/d	Test AA intake at breakpoint mg/kg/d	Max protein deposition from test AA at breakpoint %intake†	Limiting AA at breakpoint	Range of test AA intakes limited by indicator mg/kg/d
1	Zello et al 1993^(83)^	Lysine	Adults	1	14(phe)	32	5–60	37	41	Indicator	30–60
2	Kriengsinyos et al. 2002^(89)^	Lysine	Adults	1	15 (phe)	34	10–60	35	39	Indicator	30–60
3	Duncan et al. 1996^(64)^	Lysine	Adults	0·8	14(phe)	40	10–60	45	63	Indicator	30–60
4	Lazaris-Brunner et al. 1998^(125)^	Tryptophan	Adult women	1	14(phe)	32	0·5–14	4·01	33	Indicator	5–14
5	Di Buono et al. 2001^(92)^	Methionine(no cysteine)	Adults	1	14(phe)	32	0–32	12·6	32	Indicator	13–32
6	Hsu et al. 2006^(79)^	Total aromatics‡	Adults	1	45(lysine)	50	5–70	48	62	Indicator	60–70
7	Riazi et al. 2003)^(126)^	BCA§	Adults	1	15 (phe)	34	26–180	144	84	Indicator	80–180
8	Wilson et al. 2000^(127)^	Threonine	Adults	1	14(phe)	32	5–35	19	44	Indicator	20–35
9	Mager et al. 2003^(128)^	BCA§	School children	1·5	25(phe)	38	75–225	147	57	Indicator	125–225
10	Ennis et al. 2020^(82)^	Phenylalanine	Pregnant women	1·5	80(leucine), 61(tyrosine)	72	2·5–30·5	21·4	32	Test	none
11	Roberts et al. 2001^(129)^	Tyrosine	Adult men	1 (9 mg phe)	45(lysine)	(20)||	3–12	6	18	Test	6–12·5||
12	Elango et al. 2007^(124)^	Lysine	School children	1·5	25(phe)	38	5–80	41	30	Test	50–80
13	Pillai et al. 2010^(85)^	Lysine	School children	1·5	25(phe)	38	5–80	33·5	25	Test	50–80
14	Al-Mokbel et al. 2019^(130)^	Tryptophan	School children	1·5	25(phe)	38	0·5–10	4·7	26	Test	8–10
15	Turner et al. 2006^(131)^	Methionine(no cysteine)	School children	1·5	25(phe)	38	0–35	12·9	33	Test	25–35
16	Humayun et al. 2006^(132)^	Methionine (excess cysteine)	School children	1·5	25(phe)	38	0–15	5·8	15	Test	15
17	Hsu et al. 2007^(80)^	Total aromatics‡	School children	1·5	64(lysine)	47	5–70	28	24	Test	70

IAAO, indicator amino acid oxidation; EAA, essential amino acid.

* At 44 mg phe/g protein, 90·4 mg lys/g and 74 mg leu/g protein (see Table 2). Any consumption of phenylalanine to provide for tyrosine will reduce these values.

† Calculated from tissue amino acid contents in Table 2 and the test intake level at breakpoint in column 8.

‡ Phenylalanine in the absence of tyrosine.

§ 38·5, 32·5 and 29 % for leucine, valine and isoleucine, respectively.

|| Studies limited by phe intake at all tyrosine intakes above the breakpoint.

Also the test intakes did not always increase to an obvious excess, that is, ≥ levels found in egg protein. [^13^C-1]-labelled phenylalanine has been the most widely used indicator amino acid apart from studies of the requirements for phenylalanine specifically or the aromatic amino acids (AAA) when l-[^13^C-1]lysine^(79–81)^ or l-[^13^C-1]leucine was used^(82)^. Additionally, after their initial studies based on intravenous tracer administration and blood sampling^(83)^, the group developed a minimally invasive procedure involving oral administration of tracer and urinary sampling to assess amino acid tracer enrichment^(84)^, enabling application of the approach to children and pregnant women. Finally in some studies, a direct amino acid oxidation (DAAO) approach has been used, in which direct oxidation of the test amino acid is assessed such as [^13^C-1]phenylalanine to study the phenylalanine requirement^(78,82)^. This was employed even though it had been argued that with DAAO, there was concern that the precursor pool from which oxidation takes place increases as the level of the test amino acid increases, as it did with a 6-fold change in plasma phenylalanine concentrations^(82)^. It is not the intention here to examine systematically all published studies, but seventeen representative studies, ten in adults and seven in school children are reviewed in Table 1 in relation to critical aspects of the experimental design.

### Metabolic fate of the indicator amino acid intake and its potential to limit protein utilisation

As a post-prandial study, where the demand for an individual EAA always includes net protein deposition to replace post-absorptive losses and any growth, as well as other metabolic fates (see Fig. 2(b)), the relationship between the post-prandial demand and the overall maintenance requirement over 24 h is quite complex. Clearly in the piglet studies, net protein deposition for tissue growth will constitute most of the demand as recognised by the authors of those studies. Thus, the standard interpretation of indicator kinetics is that it is partitioned between oxidation and net protein synthesis as increasing test intakes improve utilisation of the dietary amino acid intake. These changes are mediated by a combination of increased insulin and amino acid levels acting to suppress protein breakdown and stimulate protein synthesis, as discussed above. However as shown in Figs. 1 and 2, PPU provides for both tissue protein deposition and metabolic consumption, both adaptive and obligatory. Which of these fates of the indicator will be most affected by a deficiency of the test EAA is a complicated question. One metabolic fate of phenylalanine as an indicator, deposition in protein as tyrosine after its hydroxylation, is usually minimised because generous amounts of tyrosine are fed to prevent this happening in all IAAO studies with this indicator. For a test amino acid such as lysine, variation in its intake is most likely to influence net protein deposition (overall and of the indicator).

Assuming, as the authors do, that protein synthesis (net deposition) to be the fate of the indicator not oxidised, the maximum extent of this which could occur can be estimated. Whilst the exact composition of post-prandial tissue protein deposition is unknown, it will occur in the splanchnic bed especially the liver, and in skeletal muscle, since in the post-absorptive state there is a net loss of muscle protein which is replaced with feeding. Table 2 shows a first approximation of the composition of post-prandial deposition as the mean values of the EAA amino acid content of porcine liver and muscle as mg/g protein. With the exception of lysine, the concentrations of all EAA in tissue protein are somewhat lower than those in the egg protein-based mixture used in most studies. These tissue values should guide any discussion of the metabolic fate of the indicator and test amino acids in the amino acid mixture fed in these studies. This is important because as indicated above, the standard design of human IAAO studies of EAA requirements involves an amino acid mixture with potentially limiting amounts of not only the test amino acid but also of the indicator.

**Table 2.** Amino acid content of tissue and egg protein

Amino acid	Tissues*	Egg†
	mg/g protein	
Ile	44	63
Leu	74	83
Lys	90	76
Met	25·6	30
Cys	13·6	22
Phe	44	55
Tyr	33·6	41
Thre	43·2	47
Trp	12	15·6
Val	52·8	70
Total BCA	171	216
Total SAA	39·2	52
Total AAA	78	96

AAA, aromatic amino acids; IAAO, indicator amino acid oxidation; EAA, essential amino acid.

* Mean of values for pork meat and liver (various animal sources) from Paul and Southgate 1978 The Composition of foods Fourth Edition^(133)^.

† Composition of the amino acid mix used in IAAO studies of EAA requirements as reported by Duncan et al.^(64)^.

The designs, and implications of the designs, for seventeen IAAO studies are shown in Table 1. Column 4 shows amino acid intakes in each study which range from 0·8 g/kg/d or, in all other adult studies, 1 g/kg/d, and 1·5 g/kg/d in children and pregnant women, respectively. Fixed indicator intakes (column 5) have been, for phenylalanine, 14–15 mg/kg/d in adult studies, and 25 mg/kg/d in studies in children and pregnant women, for lysine, 45 or 64 mg /kg/d in adults and children, and for leucine, 80 mg/kg/d in pregnant women. Each of these intakes is less (for phenylalanine much less) than what would have been supplied by the unadjusted amino acid mixture. It is usually argued that they are adequate intakes: for example, for phenylalanine, Duncan et al argue^(64)^ ‘14 mg/kg/d ensures adequate dietary phenylalanine, as previously determined by amino acid oxidation when tyrosine was present in relative excess’ quoting Zello et al. 1990^(78)^, or ‘the constant low level was given in order to ensure constant pool size’^(85)^. However, the consequence of these intakes can be objectively assessed by calculating the maximum amount of net protein deposition they would support when none is oxidised. This is shown in Table 1, column 6, as the *maximum* protein deposition allowed by the indicator (expressed as a % of the amino acid intake), for each indicator intake in column 5 on the basis of the tissue protein composition in Table 2, assuming none of it was oxidised or in the case of phenylalanine that none of it was converted to tyrosine. If this was not the case, values would be lower and the impact of limitation by the indicator would be worse. Values range from as low as 32 % for phenylalanine intakes of 14 mg/kg/d to a maximum value of 72 % when leucine at 80 mg/kg/d was used. These values can be compared with what is observed in terms of the deposition of a protein meal in healthy adults fed repeated small meals of milk protein as shown in Fig. 1; that is, of the intake of 1·2 g/kg/d, 73 % was deposited as protein. Whether these values in column 6 influence the outcome of the studies can be examined by calculating the potential amount of protein deposition allowed by the test amino acid intake at the reported breakpoint intake (the assumed requirement intake derived from the oxidation test intake plot). The range of test amino acid intakes and the intake at breakpoint are shown in columns 7 and 8, and column 9 shows the maximum protein deposition allowed by the test AA at breakpoint expressed as % intake (assuming none of the test was oxidised), calculated from the test intake level at breakpoint (column 8) and the test concentration in tissue protein (Table 2). Whether the test or indicator amino acid is limiting at the breakpoint can be examined in terms of the smaller of the values in columns 6 and 9. This shows that for the first nine studies in the table, the indicator was the limiting amino acid at the breakpoint, in some cases markedly so (studies 1, 3, 7–9) in other cases less so marked. This means that the breakpoint which would have occurred due to changes in test intake will be theoretically under-predicted, because limitation by the indicator will lower its concentration and oxidation rate, as shown by Ennis et al^(82)^, and will move the breakpoint to the left. Furthermore, in all studies listed in Table 1, at test intakes above the breakpoint, the indicator intakes were quite obviously limiting. Indicator intake limitation would have induced a low oxidation rate and determined the flat part of the oxidation plot and the breakpoint. In studies 10–17, the test amino acid was limiting below and up to the breakpoint, although with the exception of study 10 some or all of the test intakes above the breakpoint would have been limited by the indicator intake. Study 11 was a particularly complicated design since in order to study tyrosine requirements with a [^13^C-1] lysine indicator, phenylalanine intakes were limited to an extent that at all tyrosine intakes above the breakpoint, protein utilisation would have been limited by the phenylalanine intake. Only in study 10, with the leucine indicator at 80 mg/kg/d were all of the test (phenylalanine), intakes only limited by the test amino acid intake.

**Fig. 4.**
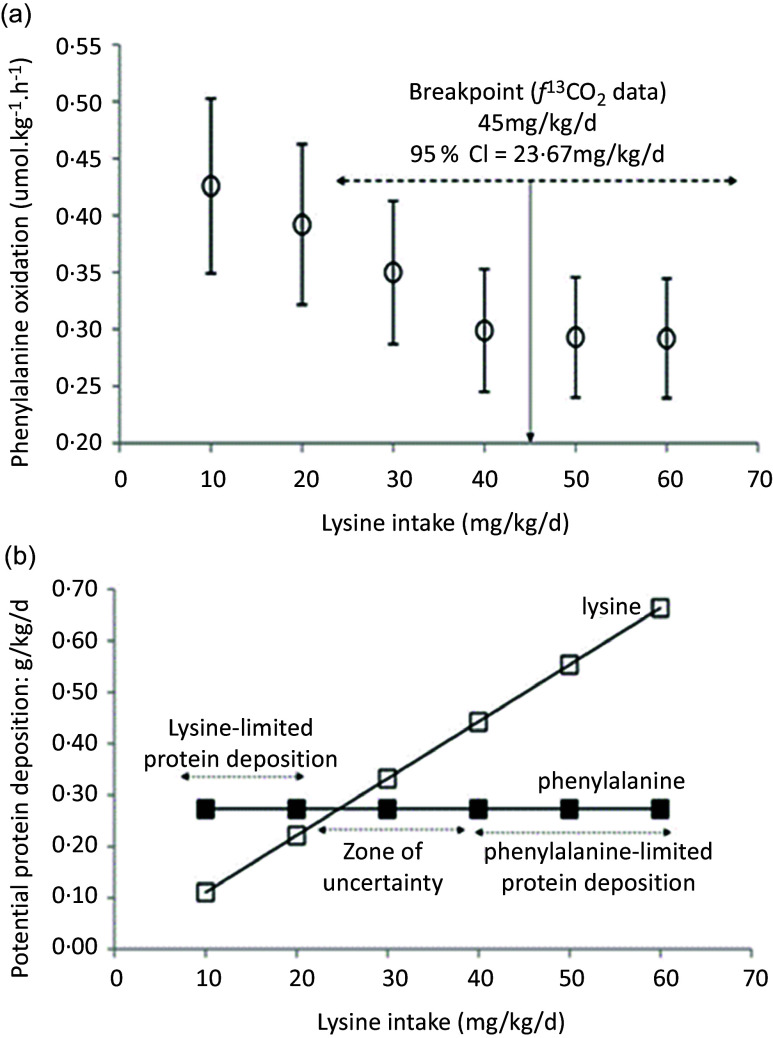
Application of the IAAO approach to measure lysine requirements in healthy adults^(64)^. (a) The influence of the dietary lysine intake on the oxidation of phenylalanine in human adults as assessed in post-prandial feeding studies. The design concept is the same as the piglet study in Fig. 3. Thus, the increasing dietary lysine intakes improves the partition of the lysine-limited dietary amino acid mixture between protein deposition and oxidation, the latter measured as oxidation of the [^13^C-1] trace-labelled ‘indicator’ amino acid, phenylalanine, until a low and constant indicator oxidation occurs after the lysine requirement is reached. This study involved five healthy adult men fed an amino acid mixture at 0·8 g/kg/d protein. Each man was studied at each of the six intakes of lysine with each study day separated by 3 d. The experimental diets were given as 6 hourly isonitrogenous and isoenergetic meals, each meal representing one-twelfth of the daily requirements with the amino acid mixture providing protein at 0·8 g/kg/d. Phenylalanine kinetics was studied with iv infusions of the ^13^C-1 phenylalanine tracer, blood sampling of the tracer enrichment during the last 4 h and ^13^CO_2_ enrichment in breath. The various lysine intakes and a fixed intakes of phenylalanine at 14 mg/kg/d and tyrosine at 40 mg/kg/d were fed with each hourly intake of a standard l-amino acid mixture based on an egg protein. The breakpoint in the *f* ^13^CO_2_ data was determined using breakpoint analysis using a two-phase linear regression crossover model. (b) In practice, protein utilisation will be limited by both lysine and the 14 mg/kg/d intake of phenylalanine. The points shown are the maximum amount of tissue protein which could theoretically be deposited from the intake if only lysine or phenylalanine were limiting, calculated assuming tissue protein contents as in Table 2, that is, lysine and phenylalanine at 90 mg/g protein and 44 mg/g, respectively. At lysine intakes ≤ 20 mg/kg/d, lysine will limit protein deposition while at intakes ≥ 40 mg/kg/d, phenylalanine will limit protein deposition with lysine intakes about 30 mg/kg/d identified as a zone of uncertainty given the unknown nature of true composition of tissue protein. Thus with increasing lysine intakes, the high phe oxidation through lysine limitation of protein deposition will fall initially as increased lysine allows increasing protein deposition, but above 30–40 mg lysine/kg/d phenylalanine will limit oxidation and oxidation will be low and constant. Had phenylalanine intakes been higher, phe oxidation may have fallen further as lysine intakes allowed further protein deposition. Thus, the limitation of protein utilisation by the indicator phenylalanine will determine the breakpoint which would be higher or may not have occurred at all if it was not limiting.

**Fig. 5.**
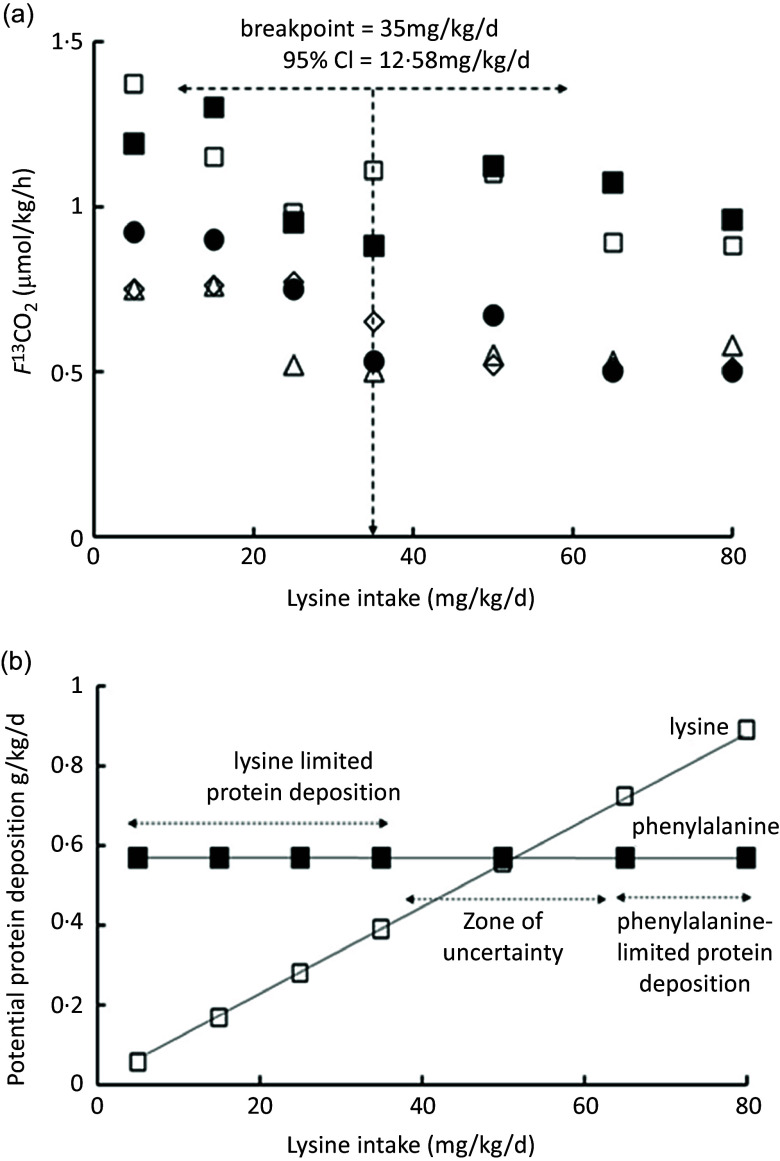
Application of the IAAO approach to measure lysine requirements in healthy children^(124)^. (a) Five 8-year-old boys and girls were fed a standardised diet with 1·5 g/kg/d protein prior to each experimental study day. Each child was studied at each of the seven intakes of lysine with each study day separated by about 1 week. On each study day, a minimally invasive IAAO study was mounted with oral administration of the ^13^C-1 phenylalanine tracer and urinary sampling of the tracer enrichment. The experimental diets were given as 8 hourly isonitrogenous and isoenergetic small meals, each meal representing one-twelfth of the daily energy requirements with the amino acid mixture providing protein at 1·5 g/kg/d, and tracer added to meals 5–8. The lysine was fed as part of a standard l-amino acid mixture based on an egg protein except for phenylalanine and tyrosine fed at 25 mg/kg/d and 61 mg/kg/d, respectively, and for lysine and alanine fed at varying intakes to maintain the isonitrogenous intakes. The breakpoint in the *f* ^13^CO_2_ data was determined using bivariate breakpoint analysis (PROC MIXED (SAS) in which choices are made between different variance–covariance structure to give the final best-fit model for the breakpoint with 95 % CI values calculated by using Fieller’s theorem. Values redrawn from Elango et al 2007^(124)^. (b) The potential for protein deposition is shown as a function of the intakes of lysine from 5 to 80 mg/kg/d, and phenylalanine at 25 mg/kg/d. As in Fig. 4(b), the points shown are the maximum amount of tissue protein which could theoretically be deposited from the intake if only lysine or phenylalanine were limiting. At lysine intakes ≤ 35 mg/kg/d, lysine will limit protein deposition while at lysine intakes ≥ 65 mg/kg/d, phenylalanine will limit protein deposition. The lysine intake at 50 mg/kg/d is identified as a zone of uncertainty given the unknown nature of true composition of tissue protein. Thus, over the intake range of lysine, indicator oxidation will be influenced by lysine at low intakes and by the indicator intake at the highest lysine intakes. Higher unlimiting indicator phenylalanine intakes would result in a breakpoint at higher lysine intakes or possibly no breakpoint at all given the upper range of lysine intakes.

Examples of these calculations for studies 3 and 12 in Table 1 are shown in Figs. 4 and 5. Figure 4(a) shows the phenylalanine oxidation data as a function of lysine intake (with the lysine intake breakpoint shown deriving from the *f* ^13^CO_2_ data), showing the wide range of the 95 % CI around the lysine breakpoint. Figure 4(b) shows the potential for protein deposition from the phenylalanine indicator and the lysine test EAA, if no other amino acid intake was limiting and no oxidation of either indicator or test amino acid occurred (any oxidation which did occur would reduce the potential for deposition shown). At the two lowest lysine intakes, lysine intake limited protein deposition, but at the three highest lysine intakes the phenylalanine indicator intakes limited protein deposition with lysine intakes of about 30 mg/kg/d identified as a zone of uncertainty, given that the true composition of tissue protein is unknown. Clearly, lysine intakes are only limiting and influencing oxidation over a minor part of the intake range. Figure 5 shows studies of the lysine requirement in children with a higher intake of the amino acid mixture and of the indicator. Figure 5(a) shows the individual *f* ^13^CO_2_ data and the reported breakpoint with the wide 95 %CIs. Figure 5(b) shows that in this case the breakpoint occurs at a lysine intake when theoretically lysine was limiting, although for lysine intakes above the breakpoint the phenylalanine indicator became increasingly limiting and would have been the main determinant of the oxidation data at these intakes, and this would markedly influence the regression analysis and breakpoint. Clearly, it is not known what proportion of the intake would have been deposited if no amino acid intakes were limiting, but in all the studies the authors state that the indicator is partitioned between oxidation and protein synthesis over the entire range of test intakes.

**Fig. 6.**
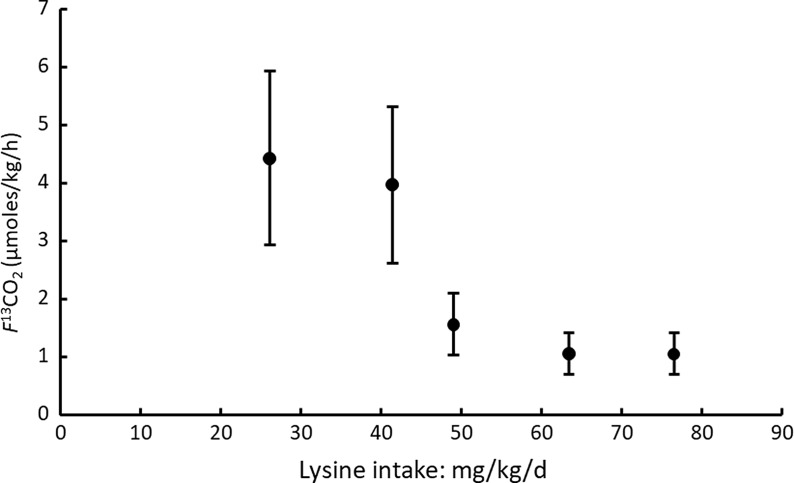
Application of IAAO approach to measure lysine requirements in healthy young male adults with unlimiting phenylalanine intakes^(86)^. This is described as a modified IAAO study in that, rather than feeding an amino acid mixture, mixed diets are fed, initially for 6 d and then on day 7, small hourly meals of the same foods were fed providing protein at 1·2 g/kg/d but with the varying lysine intakes reflecting the mix of the foods fed. This limited the lowest intake of lysine to 25 mg/kg/d. Phenylalanine and tyrosine intakes were not limiting (48 and 40 mg/kg/d, respectively). l-[1–^13^C]-phenylalanine was given orally over 4 h at the end of the 6-h feeding period, and blood was sampled once at the end of the study period. Probably as a result of the single measure of plasma phenylalanine enrichment, the reported values for the phenylalanine flux and oxidation do not appear credible, hence the use of the reported *f* ^13^CO_2_ data as shown here. Although breakpoint analysis identified a breakpoint at a lysine intake of 58 mg/kg/d, visual inspection indicates the reduction in oxidation to occur at an intake ≥ 50 mg/kg/d, equivalent to the deposition of 0·56 g protein/kg/d from the protein intake of 1·2 g/kg/d. Values redrawn from Tian et al. 2014^(86)^.

One study in which the phenylalanine intake is not limiting is reported by Tian et al. 2014^(86)^. These authors deployed what they called a modified IAAO study of the lysine requirement in that instead of an amino acid mixture, they used various Chinese food mixes, providing protein intakes at 1·2 g/kg/d, to vary the lysine contents over a 7-d adaptation period, and with small meals of chinese cabbage, pork and rice fed on the test days in seven hourly meals. The marked difference between the lysine content of cereals and animal-source foods enabled such a design, although the range of different lysine intakes was limited. Clearly, there is limited scope to study requirements for any other EAA apart from lysine with food mixtures at a constant protein intake, so this study is likely to be a one-off. Although phenylalanine intakes were not limiting at 48 mg/kg/d and the phenylalanine oxidation was measured over the last 3 hours, no evidence is given as to whether the food protein digestion and absorption was sufficient to ensure a metabolic steady state. In fact, only one blood sample was taken at the end of the last hour of the study. The attainment of a plateau ^13^CO_2_ enrichment, which was reported, does not ensure a metabolic steady state. The *f* ^13^CO_2_ results from repeated oral doses of the phenylalanine tracer is shown in Fig. 6. Although a two-phase linear regression crossover model was used to identify a breakpoint at a lysine intake of 58 mg/kg/d, visual inspection indicates the reduction in oxidation to occur at an intake between 41 and 49 mg/kg/d. Most importantly, the observed changes in oxidation could only reflect the increasing lysine intakes. Assuming tissue protein contents of lysine at 90 mg/g, the limitation of phenylalanine oxidation at about 50 mg/lysine/kg/d implies a deposition of 0·56 g protein/kg/d, a reasonable amount for these adults.

### How important is the accuracy of phenylalanine kinetics measurement?

Another difficult issue is that of accuracy of the phenylalanine kinetics. Given that the precursor for ^13^CO_2_ production from the labelled phenylalanine is hepatic tyrosine^(87)^, the values for oxidation rates, phenylalanine flux and its components, when reported, in terms of protein synthesis and proteolysis calculated from either plasma or urinary phenylalanine enrichment cannot be accurate^(69)^. The regulation of phenylalanine hydroxylation to tyrosine using enrichment in apoB-100 has been investigated with the authors concluding ‘plasma phenylalanine does not reﬂect changes in protein synthesis^(88)^. Indeed, this appears obvious from the reported values in some (but not all) studies. Thus, in the study of Kriengsinyos et al. 2002^(89)^ which investigated two tracer administration protocols for the evaluation of lysine requirements in healthy adult men with phenylalanine oxidation calculated from either the plasma or urinary phenylalanine enrichments, oxidation rates were in each case low, only a small fraction of the phenylalanine intake. The results indicated that > 75 % of the phenylalanine intake was deposited with the iv protocol and > 60 % with the oral protocol with no effect of the lysine intakes on the values and with no significant difference in phenylalanine oxidation between the lowest and highest lysine intakes with the oral protocol. These results are quite inconsistent with the basic assumptions of IAAO indicating that the true rate of phenylalanine oxidation must have been much higher. However, in some of the IAAO studies of the protein requirement discussed below^(10)^, values for the phenylalanine flux and oxidation rate are closer to what might be expected from other studies of whole-body phenylalanine kinetics^(40)^ and relatively realistic values for net phenylalanine balance can be calculated.

Does this underestimation of phenylalanine oxidation, when it occurs, matter? As argued by Mathews^(69)^ ‘It is not important in the IAAO method that the measured indicator amino acid accurately determines oxidation; rather the measured oxidation value only needs to be responsive and produce a breakpoint as a function of amino acid intake’. This may be one of the reasons that in many of these studies the oxidation data is reported only as the *F* ^13^CO_2_ the rate of ^13^CO_2_ excretion in breath, the calculation of which does not require the enrichment of the precursor pool from which it is formed. However, it is the case that if the actual precursor enrichment changes with increasing intakes of the unlabelled test amino acid, as in DAAO studies of phenylalanine requirement with a [^13^C-1] phenylalanine tracer^(78,82)^, the values for *F* ^13^CO_2_ may not be directly proportional to phenylalanine catabolism *per se.*

**Fig. 7.**
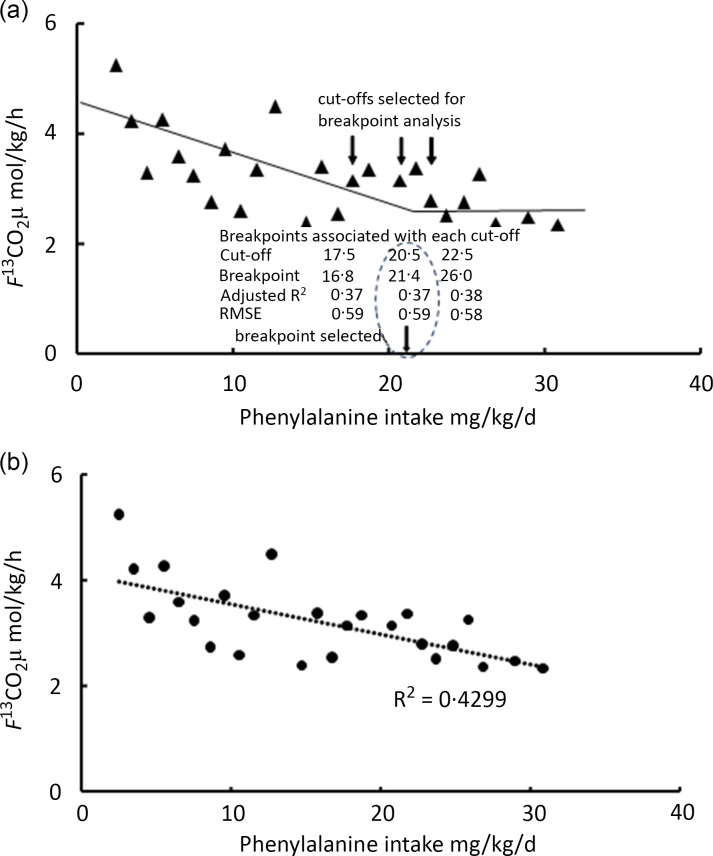
Application of biphase ‘breakpoint’ analysis to determine the phenylalanine requirements of healthy women in late pregnancy^(82)^. (a) [^13^C-1]leucine oxidation is shown in response to varying intakes of phenylalanine in women during late pregnancy (see Table 2 study 10 for the design features). The data are analysed by biphase linear regression crossover analysis. According to the authors, the analysis involves first estimating ‘cut-offs’ where the breakpoint might occur by visual inspection and then employing SAS statistical software to perform two-phase linear regression crossover analysis in order to separate the test phenylalanine intakes to the two regression lines which define the breakpoint assumed to be the phenylalanine requirement. For each initial cut-off, various models are tested with the selected one chosen for having the highest predictive ability, the lowest standard error, lowest root mean square error and the highest adjusted R^2^ value. In fact as shown in the figure, for the three cut-offs chosen, 17·5, 20·5 and 22·5 mg phenylalanine/kg/d, and for the model chosen (an unweighted-one line slope model), the three breakpoints indicated by the analysis were 16·8, 21·4 and 26·0 mg phenylalanine/kg/d and were each associated with very similar predictive abilities, R^2^ values and RMS error terms so that there was no obvious ‘best’ model. Nevertheless, the value selected, that is, 21·4 mg phenylalanine/kg/d, was similar to the value obtained in the same women by a direct oxidation protocol involving a [1–^13^C] phenylalanine tracer with increasing dietary phenylalanine intake. In this DAAO protocol, the detailed output of the statistical analysis of various models at the same cut-offs showed that breakpoints of 20–21 mg phenylalanine/kg/d were the only sensible values for any analytical model. Data redrawn from Ennis et al. 2020^(82)^. (b) An alternative analysis is shown. On the basis of the intakes of the leucine indicator and tyrosine (at 80 and 61 mg/kg/d, respectively) and their concentration in tissue protein (see Table 2), they were not limiting for protein deposition at the maximum intake of the test (phenylalanine) intake. This latter intake (30·5 mg phenylalanine/kg/d) would have allowed 50 % of the protein intake to be utilised. Thus, protein utilisation should have been driven by the test intake over the entire range with no reason for a breakpoint. Because of this from first principles, there is no reason to expect a breakpoint and the best fit of the F^13^CO_2_ data is a linear regression. This precludes identifying a phenylalanine requirement value.

The use of l-[^13^C-1] leucine has been investigated in two studies. In the first, Hsu et al. 2006^(90)^, data were reported indicating that leucine was not a good choice as an indicator amino acid for determining amino acid requirements in men. This was because in studies aimed at identifying the total AAA requirements (graded intakes of phenylalanine without tyrosine), a breakpoint could not be demonstrated when feeding leucine at 65 mg/kg/d but was demonstrated with leucine fed at a lower intake of 45 mg/kg/d. However on the basis of the calculations shown in Table 2 with leucine at 45 mg/kg/d, and the leucine and total AAA content of tissue protein shown in Table 2, leucine intakes would have become limiting for protein deposition at a phenylalanine intake of 50 mg/kg/d, so at the two highest phenylalanine intakes (55 and 65 mg/kg/d), leucine would have limited protein deposition preventing any further fall in oxidation and influencing the ‘flat’ part of the two-phase regression. In these studies, uncharacteristically at both intakes of the leucine indicator, oxidation was not highest at the lowest test amino acid intake. It was argued that this reflected ‘an inability to oxidise the large excess of leucine’ quoting a study in neonatal piglets with elevated plasma concentrations of histidine, valine, isoleucine and phenylalanine when lysine was limiting, which may indicate that the ability to oxidise the excesses of these amino acids was exceeded^(91)^ (notably, leucine concentrations were not elevated in that study). Furthermore in a second more recent study of total AAA requirements with l-[^13^C-1] leucine as an indicator fed at a higher intake of 80 mg/kg/d^(82)^ with the results shown in Fig. 7, there is no suggestion of leucine oxidation being limiting at the lowest phenylalanine intakes (as shown in Table 1 study 10) and no mention was made in this study of the potential of leucine oxidation to becoming limiting at low phenylalanine intakes. Thus, the results of Hsu et al. 2006^(90)^ remain unexplained.

### Relative insensitivity of oxidation responses in many studies and the need for breakpoint analysis

In practice, in many of these human studies, possibly as a consequence of the limitation by the indicator intakes discussed above and shown in Table 1, the demonstration of responsive oxidation rates to variation in EAA intakes with a breakpoint is much less obvious (e.g. Figs. 5 and 7) compared with the piglet studies shown in Fig. 3. When a change can be identified, the 95 % CI given to the breakpoint are wide. In some cases, it is not possible to identify the change point by visual inspection alone. For example, in a study of the requirements for the AAA with l-[^13^C-1]lysine as the indicator amino acid in five adult men fed at eight intakes of phenylalanine without tyrosine,^(79)^ the variability in the individual responses is so great that there is no significant difference between mean values for the *F* ^13^CO_2_ at the lowest and the highest phenylalanine intake. Indeed for one subject (subject 3 in figure 2 of that paper), the dose–response curve is essentially flat. In studies of the total *S*-amino acid requirements as methionine apart from higher phenylalanine oxidation with zero methionine intakes, oxidation rates did not change significantly with any intake of methionine^(92)^. Similarly in the comparison of iv and oral protocols for the evaluation of lysine intakes in healthy men referred to above^(89)^, phenylalanine oxidation was not different between the lowest or highest lysine intakes in the oral isotope protocol, and no obvious breakpoint was apparent with the iv isotope protocol, most likely as a result of the limitation of the indicator intake at the breakpoint and at all higher lysine intakes shown in Table 1. Nevertheless in this latter study, as with all other published IAAO studies, a complex specific statistical analysis was deployed to analyse the data, biphase linear regression crossover analysis, with zero slope assumed for the highest intakes of the aggregated data of all subjects on all intakes.

Reliance on the two-phase analysis in these studies is such that in a study which examined phenylalanine oxidation in subjects fed lysine at four different intakes (5, 20, 35 and 70 mg/kg/d) for different periods of adaptation (8 h, 3 d and 7 d)^(93)^, which showed reduced and more or less constant oxidation at lysine intakes of 20 mg lysine kg/d and higher, no conclusion about the implications for the lysine requirement was drawn. The authors argued that with only four intakes, no statistical analysis could be applied, commenting ‘It is unclear why the actual *f* ^13^CO_2_ is lower on a deﬁcient intake (20 mg/kg/d), although in requirement studies where relative rates of *f* ^13^CO_2_ are compared over a range of 6–7 test amino acid intakes, the actual *f* ^13^CO_2_ at an individual intake level does not inﬂuence the breakpoint analysis’. In fact in this study in which 15 mg/kg/d phenylalanine was fed, at the two highest lysine intakes the intake of the phenylalanine indicator was limiting, as in other lysine IAAO studies showed in Table 1 ^(64,83,89)^. This would have prevented any fall in indicator oxidation at the two highest lysine intakes which would have been suggestive of a higher lysine requirement. It is the case that in 24-h ^13^CO_2_ leucine oxidation balance studies of the lysine requirement in healthy Indian subjects, at intakes of 20 mg lysine/kg/d subjects were not in balance, with 28 mg/kg/d required for overall leucine balance (Kurpad et al. 2002)^(94)^.

Further insight into the use of statistical breakpoint analysis in these IAAO studies is described in some individual studies (e.g. Pillai et al 2010^(85)^ and Ennis et al. 2020^(82)^). Also, Hayamizu and co-workers have written extensively about the theoretical basis of change point analysis of IAAO data (Hayamizu et al. 2011^(95)^). Their work includes accounting for individual variability^(96)^ and adjusting for any isotopic carry-over effect^(97)^ (but no comments are made about the potential influence of indicator intake limitation of the oxidation). In the study of the lysine requirement in well-nourished Indian children^(85)^, the authors describe the use of PROC MIXED (SAS/STAT version 8.2, SAS Institute in which choices are made between different variance–covariance structures to give the final best-fit model and breakpoint. The most complete description occurs in a recent study of the phenylalanine requirement in pregnant healthy women^(82)^. In this study, both the DAAO approach with [^13^C-1] phenylalanine and an IAAO approach with [^13^C-1] leucine are deployed, with the latter study shown in Fig. 7(a) and Table 1 (study 10). In order to apply the biphase linear regression crossover analysis, it is explained that cut-offs in the dose–response data must be first chosen, presumably on the basis of visual inspection, which are then subject to a number of different analytical models, that is, whether only one or both regression lines had slopes and whether the regression lines were weighted or unweighted. Thus for each chosen cut-off, four models were analysed producing four breakpoints, each with its adjusted R^2^ values, CV, RMS standard error and predictive ability. In fact, according to the reported analytical outputs (shown in online Supplementary Tables), the three cut-offs chosen (see Fig. 7(a)) 17·5, 20·5 and 22·5 mg phenylalanine/kg/d indicated breakpoints of 16·8, 21·4 and 26·0 mg phenylalanine/kg/d, and each were associated with similar R^2^ values, and high RMS error terms, that is, no obvious ‘best’ model. Since the DAAO study with [^13^C-1] phenylalanine indicated an obvious breakpoint of 21 mg/kg/d, with a large R^2^ and low CV and RMS se, it is possible that this result guided their [^13^C-1] leucine oxidation breakpoint selection. This suggests that IAAO breakpoint analysis is by no means objective. In fact as shown in Fig. 7(b), the best statistical fit with no a priori assumptions is a linear fall in leucine oxidation over the entire range of intakes. This suggests that at the highest phenylalanine intake (30 mg/kg/d), the phenylalanine was not in excess. 30 mg/kg/d phenylalanine would have allowed the deposition of 0·75 g of tissue protein on the basis of the tissue composition in Table 2, that is, utilisation of 50 % of the amino acid mixture intake. This is assuming that the amino acid mixture was perfectly balanced and that the MD for PPU allowed such a deposition in these late gestational pregnant women^(82)^.

### Conclusions on human indicator amino acid oxidation studies of essential amino acid requirements

The above discussion indicates that IAAO studies of EAA requirements in growing animals are theoretically sound and conform with the principles described by David Baker^(24)^ for the evaluation of the EAA requirements for growth. EAA requirements in healthy adults are for maintenance as are most of the requirements in children and pregnant women, and there is no obvious reason why IAAO should not be able to measure that and provide results similar to the 24-h [^13^C-1] leucine balance studies of amino acid requirements conducted by Kurpad and Young^(94,98)^. However, the analysis of IAAO studies of EAA requirements in Table 1 identifies serious constraints in their design and consequent concerns about the validity of their findings. The piglet studies shown in Fig. 3 demonstrate the design features necessary to produce valid breakpoint features of indicator oxidation responses to varying test EAA intakes: (a) meal protein utilisation limited only by intakes of the test EAA; (b) test intakes fed from obviously deficient to an obvious excess; and (c) an indicator of which its oxidation can be assessed through capture of labelled CO_2_. With these constraints, the breakpoint should correspond to an intake of the test amino acid which allows maximal utilisation of the food intake, that is, an intake meeting the MD for the test EAA, that is, its requirement. In human nutrition, no formal definition of the requirement for an EAA *per se* is given in the WHO report^(1)^, but it can be assumed to be an amino acid intake which allows the maintenance of the lean body mass in healthy well-nourished adults of all ages and which provides for any special needs. In the context of the metabolic model in Fig. 2, it is the EAA intake which meets the post-prandial demand at an intake providing the protein requirement of the subjects. However, none of the studies listed in Table 1 satisfied the first two criteria identified above in relation to the piglet study. With the single exception of study 10 shown in Fig. 7 ^(82)^, all seventeen studies involved intakes of the indicator which at some level of test intake became more limiting than the test intake: nine were limited by the indicator at the breakpoint and eight at one or more of the higher test EAA intakes. Second, only in one case did the range of test intakes extend to an obvious excess within the amino acid mixture allowing a true minimisation of indicator oxidation. These two factors alone mean that any breakpoint related to the ‘required’ intake of the test amino acid would have been under-predicted. Whether the design limitations identified in Table 1 contribute to the lack of sensitivity of changes in phenylalanine oxidation in many of the studies is difficult to determine. In any event, their low sensitivity inevitably appears to result in a subjective analysis (at least in the one study in which the breakpoint analysis has been described in detail). Statistical analysis is usually employed to determine whether observed differences between values obtained in a study are actually different rather than in this case to identify differences which are not observed. It is also arguable that for many investigators, phenylalanine would not be the indicator of choice. However, this issue is probably less important than the difficulties associated with limiting intakes of the indicator. In theory, well-designed human IAAO studies conducted after full adaptation to the protein requirement intake should reflect the maintenance requirement. As shown in Fig. 2(b), this includes the demand for some net protein deposition – according to both the extent of any recycling^(20,52)^ and the amplitude of gains and losses – together with that of the adaptive and obligatory oxidative demands. Nitrogen or ^13^C carbon balance studies over 24 h or longer in fully adapted adults should identify the same maintenance requirement, and these have also been conducted with amino acid mixtures. It must be assumed that the explanation for the 24-h balance studies resulting in lower values (e.g. for lysine) than IAAO studies published to date reflects some of the design and analysis features discussed in this section. It remains to be seen whether well-designed human IAAO studies conducted after full adaptation to the protein requirement intake would also result in lower values. Some aspects of the incomplete adaptation issue are identifiable in the next section on IAAO studies of the protein requirements.

## Indicator amino acid oxidation studies of the protein requirement: background

Coincident with the publication of the WHO 2007 report, the first use of IAAO to determine protein requirements was reported^(10)^. Since then, several further reports in various human population groups have been reported by the group, and the method has been adopted by others (see Matsumoto et al for a recent listing of all studies^(65)^). Although it has been argued that the use of the IAAO approach to measure protein rather than EAA requirements is fundamentally flawed^(99,100)^, the authors dispute such criticisms^(101,102)^. In some IAAO studies of the protein requirement, they state ‘According to the Institute of Medicine, the IAAO technique is an acceptable method to assess protein requirements’^(103)^ quoting the 2005 IOM DRI report^(3)^. However, that report made no mention of the use of IAAO to assess protein requirements, since no such human study had been published at that time. Furthermore in the recent scoping review of ‘Evaluation of protein requirements using the IAAO method’^(65)^, the methodology itself was not discussed: indeed, the use of IAAO to assess protein requirements has not previously been critically evaluated, or fully explained by its authors. In its first application for this purpose, Humayun et al., 2007^(10)^, the authors say little about the theoretical basis of this application other than ‘The IAAO is a robust technique that has been successfully used previously by our group to determine protein requirements in pigs and by our group and others to determine amino acid requirements in adults and children’ and ‘This is first study that used the IAAO technique to determine protein requirements in healthy adults. Previously, Ball and Bayley used the IAAO technique and estimated protein requirements for growing piglets’. In a review of the method in 2012^(16)^, it was argued ‘The intake of phenylalanine (indicator amino acid) was maintained constant, with excess tyrosine, to ensure that with increasing intakes of total protein nitrogen the indicator amino acid was partitioned between oxidation and protein synthesis’. Whilst this would account for a fall in oxidation as the increasing total protein nitrogen allowing increasing deposition of the indicator, how or why a breakpoint was reached and its relationship with the protein requirement was never explained. Because the results in terms of the breath ^13^CO_2_ *v*. protein intake curve appear to be similar to results obtained in the EAA requirement studies, casual readers will assume that the experimental protocol is the same. However, the paradigm is different and quite different considerations apply. Also, because the original piglet studies are the only studies of this particular application of IAAO in which the group make any attempt to explain and further investigate the experimental design, it is instructive to examine that study^(104)^, especially in the context of what might be expected of post-prandial amino acid oxidation in response to different levels of protein intake.

### What might be expected in multi-level post-prandial protein intake studies?

From the outset, there is a difficulty in designing a protocol to identify the protein requirement during multilevel post-prandial protein feeding studies based on the partition of an indicator amino acid between oxidation and net protein synthesis. As discussed above in relation to Fig. 1, overall amino acid oxidation and nitrogen excretion does not change much between the post-absorptive state and repeated small meals of a low followed by a moderate protein intake. This is because overall amino acid oxidation would be expected to be low at all levels of protein intake up to the level which can no longer be deposited and then oxidation would increase. This pattern of response has in fact been reported. For example, the responses of leucine oxidation to increasing protein intakes over a range of intakes in two similar studies of Chinese adults have been reported^(105,106)^. In each case, leucine oxidation was measured during hourly feeding of small meals after 7 d of diets with varying levels of protein intakes showing that at protein intakes between 0·8 and 1 g/kg/d, leucine oxidation did not change but increases occurred at intakes of 1·1 and 1·2 g/kg/d in the first study and above 0·98 g/kg/d in the second study. This was presumably because at these higher intakes, protein utilisation for deposition was incomplete.

Moore and colleagues have examined the response to increasing protein intakes in post-exercise studies in children focusing on the effectiveness of protein meals to achieve an overall anabolic response. They examined [^15^N] kinetics with [^15^N]glycine by means of the [^15^N]ammonia end product^(107)^, and [^13^C-1]leucine oxidation, turnover and balance^(108)^. In each case, higher intakes of protein improved overall N or leucine balance. In the latter study after a single meal of increasing intakes of skim milk protein (0, 5, 10 and 15 g, that is, up to half their daily requirement), [^13^C-1] leucine balance increased linearly with increasing protein intake with leucine oxidation increasing to a plateau at the two highest intakes.

Taken together these results show that during post-prandial studies of protein utilisation and amino acid oxidation, a response curve relating amino acid oxidation to protein intake will not reveal information about the protein requirement other than possibly the changes in protein utilisation as intakes increase, if this occurs.

### Development of the indicator amino acid oxidation approach to protein requirements in the piglet

**Fig. 8.**
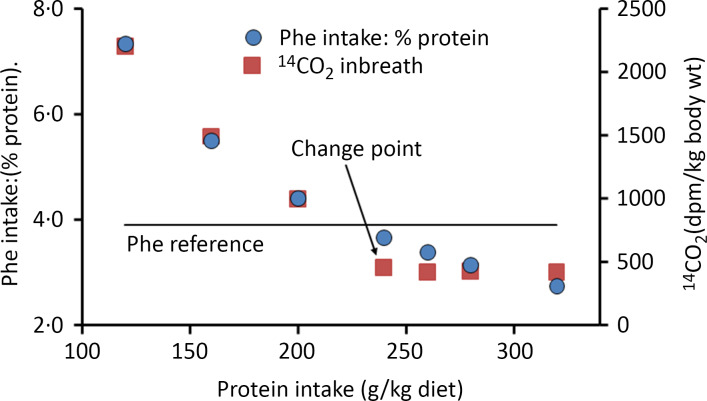
Design and results of first use of the IAAO method to measure protein requirements in young pigs^(104)^. Piglets were fed two liquid meals containing skim milk plus various levels of an amino acid mixture patterned on skim milk but without phenylalanine or tyrosine and with trace amounts of [1–^14^C] phenylalanine. Variable amounts of phenylalanine and tyrosine were added to each meal to ensure a fixed absolute intake of these two amino acids for all levels of protein intake so that their intake was equivalent to that provided by 200 g protein/kg feed. Thus, the dietary phenylalanine concentration was very high at the lowest protein intake and very low at the highest with values shown here as a % of the protein fed at each protein intake. The extent of this excess or deficiency can be observed in comparison with the phenylalanine reference which is the concentration of phenylalanine in pig tissue proteins (3·9 % protein^(109)^), which as discussed in the text is a minimum value for the phenylalanine requirement in these growing pigs. Breath CO_2_ was collected over 60 min following the second meal. Phenylalanine oxidation, as indicated by ^14^CO_2_ in breath, was high at low-protein intakes as the excess phenylalanine was oxidised, the rate decreasing as the intake become more balanced, falling to a low constant level at 240 g protein/kg feed at which level the phenylalanine content of the feed matched that in the deposited tissue. This ‘change point’ for phenylalanine oxidation was identified as the protein requirement level. At intakes above 240 g/kg diet phenylalanine and tyrosine were limiting for protein deposition, so phenylalanine oxidation fell further eventually reaching a low constant level. Measurement of ^14^C uptake into liver protein in the piglets showed that at intakes up to 240 g/kg diet, most of the dietary protein was utilised for protein deposition but at higher intakes ^14^C uptake into liver protein did not increase further demonstrating the limitation of phenylalanine and tyrosine for tissue protein synthesis. Figure drawn with data from original study^(104)^.

Ball and Bayley^(104)^ modified IAAO to obtain a response curve to varying protein intakes which was similar to that observed in IAAO studies with limiting EAA, that is, quite different to those studies discussed above in the previous section. This was achieved by feeding an amino acid mixture as part of their protein intakes, enabling phenylalanine (and tyrosine) intakes to be kept constant while varying the overall amino acid and protein intake. They stated ‘… the oxidation of [^14^C]phenylalanine with increasing dietary protein concentration, based on the response of the pig to the most limiting amino acid, should be useful for determining the dietary protein concentration required to maximise tissue deposition of the dietary amino acids’, without explaining how this would be achieved. They adopted a complex dietary design of fixed or variable amounts of skim milk fed with fixed or variable amounts of an amino acid mixture formulated without any phenylalanine or tyrosine to give an overall range of protein intakes between 120 and 320 g/kg diet. To each intake level, they added phenylalanine and tyrosine to ensure a fixed constant dietary intake (8·8 g/kg phenylalanine and 8·5 g/kg tyrosine: the amount present in 200 g of milk protein). The authors argued that ‘the concentration of total and free phenylalanine was maintained constant in all the diets to avoid a variable dilution of the [^l4^C-1] phenylalanine with dietary phenylalanine’. Figure 8 shows the design in terms of phenylalanine intake as a % of the overall protein–amino acid mixture in relation to a ‘reference’ intake of phenylalanine which is the % content of phenylalanine in pig carcass protein (3·9 % protein^(109)^), and the phenylalanine oxidation as *f* ^13^CO_2_ at each level of intake. It is clear that phenylalanine (and tyrosine) intakes were either in excess, to a variable degree at intakes below 240 g/kg diet, or insufficient to allow complete protein utilisation, had this been possible, at higher intakes. On this basis, there would have been a 100 % excess at the lowest protein intake and a deficiency ≥ 30 % at the highest intake, with the phenylalanine oxidation reflecting this. Any additional MD for phenylalanine for metabolic purposes could increase the magnitude of the reference intake and the extent of deficiency at the higher intakes. One way of expressing the consequences of the fixed phenylalanine intake is in terms of protein quality of the intake: the amino acid score of the intake would have fallen at protein intakes > 240 g/kg/d, to about 0·7 at the highest intake. Thus, even though the phenylalanine intake has been described as the ‘requirement’ level^(101)^, the phenylalanine intake was less than that required for efficient utilisation of the highest protein intakes for tissue protein deposition. This explains the shape of the ^14^CO_2_ excretion curve in Fig. 8, similar to the shape of the curve in Fig. 3. However while in Fig. 3, the phenylalanine oxidation is acting as an indicator of the oxidation of all other amino acids whose utilisation is limited by the test amino acid (tryptophan), as shown in Fig. 8, phenylalanine oxidation is only indicating its own excess or limitation for post-prandial protein deposition. The change point reflects mainly the design, that is, formulating the meals to contain phenylalanine + tyrosine equivalent to 200 g/kg of milk protein. In fact, in these pig studies, this limitation for protein deposition at the highest intakes was shown by the authors as tracer uptake into liver protein. This increased with increasing intakes up to the change point in phenylalanine oxidation but did not increase further at intakes above it. The authors did acknowledge this consequence of their design to some extent by stating that ‘the relative excess of phenylalanine would decrease with increasing dietary concentration of the limiting amino acid, providing phenylalanine for oxidation in inverse proportion to the amount taken up for protein synthesis’, which is correct as far as it goes. The change point in the ^14^CO_2_ oxidation curve was identified as the protein requirement level, that is, ‘These results extend the application of the oxidation of an indicator amino acid to the determination of the response of piglets to increasing dietary protein concentration, providing a clear conclusion as to the optimum protein level in the diet by making observations on piglets at a single weight, growing at a uniform rate’. It was also stated ‘The changes in phenylalanine oxidation with increasing protein concentration show that fractional phenylalanine oxidation may be responsive to changes in protein retention’. Whilst this is correct, the responsiveness is due to the reduction of the excess of phenylalanine with increasing protein concentration. The ‘optimum level of protein in the diet’, the change point, reflected the intake within which phenylalanine concentration matched that of tissue deposited. It is difficult to conceive how the change point provides any information about overall protein requirements or protein deposition which is independent of the experimental design.

### Application of the indicator amino acid oxidation approach for the study of human protein requirements: potential limitation of phenylalanine intakes

**Fig. 9.**
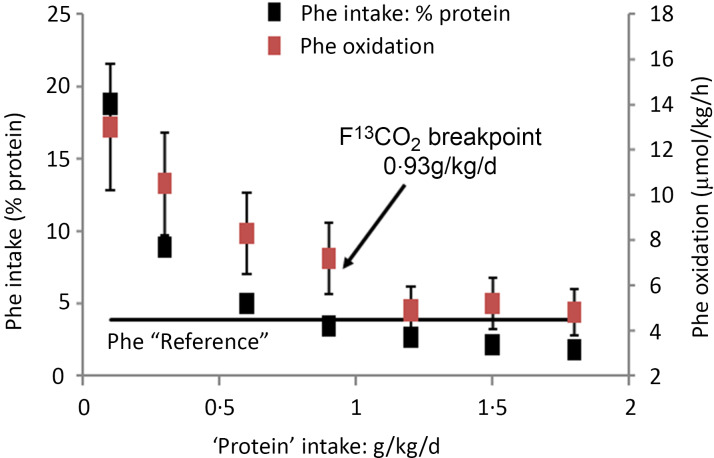
Design and results of an IAAO study of protein requirements of young men^(10)^. This was the first application of IAAO for the assessment of protein requirements in humans. Subjects were fed hourly meals containing increasing amounts of the amino acid mixture patterned on egg protein but containing fixed amounts of phenylalanine (30·5 mg/kg/d) and tyrosine (40·7 mg/kg/d). Oral doses of l-[1–^13^C] phenylalanine were given with the last 3 of 8 successive hourly meals, and phenylalanine oxidation rates were derived from ^13^CO_2_ excretion in breath and urinary l-[1–^13^C]phenylalanine enrichment. Thus, the phenylalanine intake as % of the amino acid mixture fed was in marked excess at intakes below 0·9 g protein/kg/d but was in deficit at higher intakes which is apparent by comparison with the phenylalanine reference, the concentration of phenylalanine in the tissue proteins which would have been deposited (4·4 %, Table 2). On this basis, the 30·5 mg fed would limit protein deposition to a maximum of 0·69 g/kg of tissue protein and at higher intakes no further protein deposition would occur and phe oxidation would stay low and constant. Biphase linear regression crossover analysis of the *F* ^13^CO_2_ excretion–protein intake relationship indicated a breakpoint as shown at 0·93 g/kg/d.

This experimental design was first used in 2007 to assess human protein requirements in young men^(10)^. The study design involved repeated small meals of an amino acid mixture patterned on egg protein which contained a ﬁxed intake of phenylalanine and tyrosine at 30·5 and 40 mg/kg/d, respectively, and with oral doses of l-[^13^C-1] phenylalanine given with the last 3 of 8 successive hourly meals. Figure 9 shows the design in terms of phenylalanine intake, as a % of the amino acid mixture in relation to a phenylalanine ‘reference’ intake, its concentration in tissue protein (4·4 % protein, Table 2), and phenylalanine oxidation with increasing ‘protein’ intake. As with the piglet studies shown in Fig. 8, the constant absolute phenylalanine intake resulted in changes in the intake from marked excess of phenylalanine at the lowest to severe limitation at the highest protein intakes. The phenylalanine content of the intake changed from 20 % to only 1·8 % at the highest intake, providing < 50 % of the amount needed to allow complete utilisation of the highest intake. The 30·5 mg phenylalanine fed would have allowed a maximum of 0·69 g/kg of tissue protein to be deposited. The phenylalanine oxidation pattern reflects this excess and deficit falling from a high rate at the lowest intake to a low and constant level at intakes > 0·9 g/kg/d. Bilinear regression identified a breakpoint in the *F* ^13^CO_2_ data at 0·93 g/kg/d. Thus, none of the three highest amino acid intakes could have been efficiently utilised because of the phenylalanine limitation so that overall amino acid oxidation would have increased, but this is not shown with this design because the indicator is not showing overall amino acid oxidation. This was demonstrated in studies using an identical design to this with various groups of adult and adolescent females and men after exercise fed meals of increasing protein up to high levels with measurements of the urinary urea:creatinine ratio^(110–112)^. This increased linearly with increasing intakes.

The design shown in Fig. 9 has been used in most subsequent studies by these authors and by other research groups (e.g. Daniel Moore’s group^(110–113)^ with the exception of studies performed with Peter Lemon’s group which adopted a lower intake of phenylalanine, at 25 mg/kg/d (with no explanation)^(103,114)^. As already indicated, Matsumoto et al. 2023 has given a complete listing of use of the IAAO method to asses protein requirements^(65)^. Some subsequent studies adopting this same experimental approach in subjects with higher habitual protein intakes up to 3·0 g/kg/d (e.g. Mazzulla et al. 2020^(99)^, or 3·5 g/kg/d^(103,114)^) would have involved intakes at the highest level which were grossly deficient in phenylalanine (and tyrosine), that is, phenylalanine at only 1·1 % of the amino acid mixture at 3·0 g/kg/d,^(110–113)^, and only 0·7 % of the 3·5 g/kg/d fed with only 25 mg/kg/d phenylalanine^(103,114)^.

The identification of phenylalanine as limiting for protein deposition at high ‘protein’ intakes, shown in Figs. 8 and 9, is the most contentious issue about the application of the IAAO approach to assessing protein requirements. In response to such an analysis^(99,100)^, the authors argued ‘We have previously measured the phenylalanine requirement in the presence of an excess of all other amino acids (i.e. protein), including tyrosine, and found it to be 13·6 mg/kg/d^(78)^. During the present experiment, we provided phenylalanine at an intake of 30·5 mg/kg/d, which is well in excess of the phenylalanine requirement in the presence of excess tyrosine (40 mg/kg/d) and other amino acids. Therefore, very clearly, phenylalanine would not be limiting at any intake of protein in the current experiment’^(102)^. Given that 30·5 mg phenylalanine can only allow a maximum of 0·69 g tissue protein to be deposited, and any use of the dietary phenylalanine for metabolic purposes would reduce the amount of protein which could be deposited, this is simply not the case. While the values shown in Table 1 for the phenylalanine concentration in human tissues can only be considered a first approximation actual composition of human post-prandial protein deposition shown in Fig. 9 and the value for the pig in Fig. 8 is a measured value for pig carcass protein, the ‘reference’ intakes shown are unlikely to be different from actual values. This means that intakes ≥ 240 g/kg diet in the piglet studies (Fig. 8), ≥ 0·9 g/kg/d in human adults (Fig. 9), and > 0·6 g/kg/d in the studies of bodybuilders and endurance athletes studied with the lower fixed intake of 25 mg/kg/d of phenylalanine will be indisputably limited by their phenylalanine content explaining the low plateau oxidation rate and constant net phenylalanine balance at high intakes.

It is the case that in all of the reviews of this application of the IAAO approach, the fate of the dietary protein in terms of protein deposition is never discussed^(16,63)^. This is in contrast to the studies of Daniel Moore’s group in which their objective is to maximise net protein deposition after exercise, for example, Mazzulla et al. 2020^(110)^, and they adopt the IAAO protocol with increasing intakes of the amino acid mixture with the fixed phenylalanine intake of 30·5 mg/kg/d which they specifically describe as an excess ‘to ensure metabolic partitioning of the phenylalanine carboxyl group to either synthesis or oxidation’, quoting Zello, Pencharz and Ball 1990^(78)^ as their justification. In their studies at the highest intakes of 3 g/kg/d of the amino acid mixture, in order for this to be efficiently utilised for tissue protein deposition, it would have to contain > 100 mg phenylalanine.

**Fig. 10.**
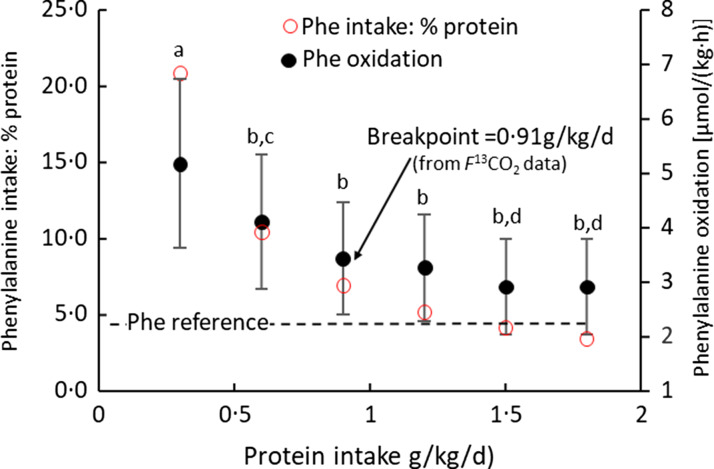
Design and results of an IAAO study of protein requirements of Chinese elderly adults with unlimiting phenylalanine intakes^(66)^. Subjects were fed 8 hourly meals containing increasing amounts of lactalbumin together with additional amounts of phenylalanine to maintain a fixed intake at every protein intake of 62·8 mg/kg/d phenylalanine and 61·4 mg/kg/d tyrosine, the level provided by the highest intake of lactalbumin (1·8 g/kg/d), twice the level in the study shown in Fig. 9. As shown, this meant that phenylalanine intake was in marked excess at the lowest protein intakes, but the intake became balanced at the two highest intakes similar to the reference intake, the phenylalanine content of tissue protein as indicated in Table 2. Oral doses of l-[1–^13^C] phenylalanine were given with the last four of the eight successive hourly meals, and phenylalanine oxidation was calculated from ^13^CO_2_ excretion in breath and urinary l-[1–^13^C] phenylalanine enrichment. Phenylalanine oxidation fell with increasing protein intakes, as the excess phenylalanine intake fell, reaching the lowest level with the two highest protein intakes. A non-linear mixed-effects model regression analysis was applied to the pooled *F* ^13^CO_2_ data for all subjects which identified a breakpoint at a protein intake of 0·91 g/kg/d. However, as discussed in the text, no breakpoint is apparent.

The question of what responses would be observed in these studies of the protein requirement with a non-limiting intake of phenylalanine can to some extent be examined from a recent application of the IAAO method in a study of protein requirements of Chinese elderly adults. In this study^(66)^, the minimally invasive procedure (oral administration of tracer and urinary sampling for phenylalanine tracer enrichment) was adopted in an experimental design which differed from previous applications in two ways. First rather than use of an amino acid mixture, the authors used varying intakes of lactalbumin protein (from 0·3 to 1·8 g/kg/d). As a result, every level of intake could have, at least in theory, been fully utilised for post-prandial protein deposition. Second and most importantly, in order to keep phenylalanine and tyrosine intakes constant, additions of these two amino acids were made so that their total intakes (from the protein and the additions) were equal to 62·8 mg/kg/d phenylalanine and 61·4 mg/kg/d tyrosine, the level provided by the highest intake of lactalbumin, that is, twice the level used in most previous studies. As shown in Fig. 10, the phenylalanine content of the intakes fell from > 20 % protein, a 5× excess, at the lowest protein intake to that provided just by lactalbumin at the highest intake. This allowed phenylalanine oxidation responses to be examined without any intakes limited by insufficient phenylalanine.

Whilst there was considerable variability in phenylalanine oxidation with phenylalanine flux and oxidation rates lower than expected (compare oxidation rates in Fig. 10 with Fig. 9), which the authors comment on, the rate fell more or less continuously with increasing protein intakes reaching the lowest level with the two highest protein intakes which were significantly lower than the two lowest protein intakes. How would rates of phenylalanine oxidation be predicted to change with this experimental design of unlimiting phenylalanine intake? Phenylalanine oxidation rates would be influenced by (a) the extent of any adaptive MD at all intakes increasing oxidation, (b) the extent of the excess intake relative to the reference intake (tissue phenylalanine content), increasing oxidation: this excess, as shown in Fig. 10, decreases with increasing intakes over the entire intake range, and (c) the efficiency of protein utilisation for tissue protein deposition, which can be assumed to be high at the lowest protein intakes but which is unknown at the highest intakes which may approach or exceed the upper limit for deposition in these subjects and could increase oxidation. Given the large between-subject variation, significant changes in oxidation between individual protein intakes would only expected to be observed with large differences in the relative excess phenylalanine intakes, as with the three lowest intakes. As for the highest protein intakes, previous l-[^13^C-1] leucine oxidation studies of Chinese adults fed increasing protein intakes^(105)^ and^(106)^ with increasing protein intakes reported oxidation began to increase (i.e. utilisation began to decrease), at intakes > 0·9–1 g/kg/d in each study. If this also occurred with the highest lactalbumin intakes in the studies shown in Fig. 10 ^(66)^, then the falling oxidation due to the reduction in the ‘excess’ phenylalanine intake would be to some extent balanced by an increasing oxidation due to a reduced efficiency of utilisation. At the highest intake with no additional phenylalanine intake, phenylalanine oxidation and net balance could be estimated if the kinetic data were accurate. The fact that there is no indication that oxidation actually increased at the highest intakes suggests that these two opposing factors to some extent cancelled each other out. Clearly, this design does not force the higher intakes to be associated with a low and constant oxidation rate due to phenylalanine limitation. However, given the overall shape of the phenylalanine oxidation–protein intake relationship, the result of the non-linear mixed-effects model regression analysis of the pooled *F* ^13^CO_2_ data which the authors deployed to identify a breakpoint at a protein intake of 0·91 g/kg/d is not surprising. Assuming the authors did not define a cut-off in their analysis (as discussed above in relation to Fig. 7, but of which no mention is made in this study), simply instructing the analysis to partition the data between two lines with at least three intakes to each line and with the second line defined as having zero slope would be expected to identify the breakpoint intake close to 0·9 g protein/kg/d because of the way in which the application of this analysis works in practice. At no point in this report is any rationale given for the experimental design other than stating ‘Indicator amino acid oxidation (IAAO), which was initially used to study amino acid requirements, is a robust method for calculating protein requirements by measuring changes in the oxidation of a labelled amino acid’, and ‘phenylalanine and tyrosine intake amounts were kept constant at 62·8 mg/(kg·d) and 61·4 mg/(kg·d), respectively, higher than in the studies previously mentioned^(66)^’.

### Application of indicator amino acid oxidation to assessment of protein utilisation during post-exercise anabolism: influence of background diets on outcomes

**Fig. 11.**
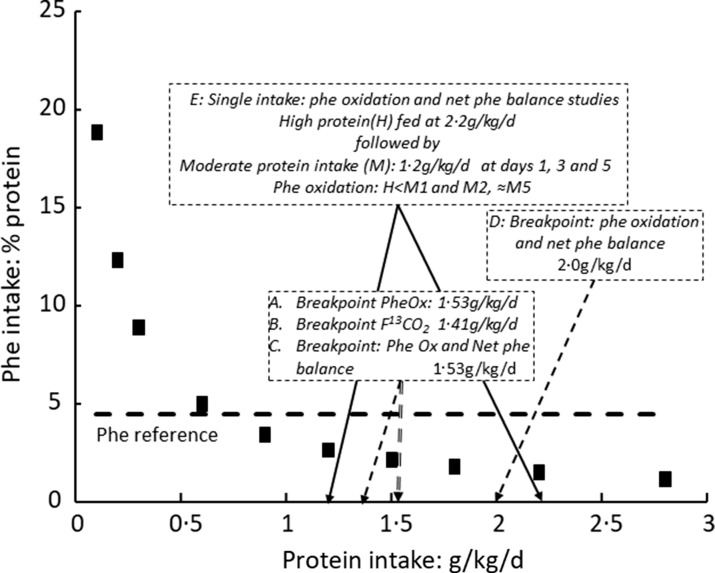
Design and results of IAAO studies of post-exercise anabolism. This shows the range of intakes of the amino acid mixture modelled on egg protein containing fixed intakes of phenylalanine (at 30·5 mg/kg/d) and tyrosine (at 40 mg/kg/d) fed, in each of five reports from Daniel Moore’s group of post-prandial, post-exercise studies of anabolism, involving the minimally invasive ^13^C phenylalanine IAAO approach, together with the study results mainly in terms of protein requirements. The phenylalanine intakes shown here as a % of the overall amino acid intakes varied from a marked excess at the lowest intakes to a marked deficiency at the highest intakes in comparison with a reference value, the phenylalanine content of tissue protein. The studies indicated in the boxes are as follows. (a) Protein requirements in endurance athletes after exercise with breakpoint analysis of the phenylalanine oxidation over the range of intakes shown here with the breakpoint at 1·53 g/kg/d^(113)^. (b) Protein requirements of female athletes measured after variable intensity exercise with a breakpoint at 1·41 g/kg/d^(115)^. (c) The protein intake required to maximise whole-body anabolism in resistance-trained females after exercise with breakpoint analysis of both phenylalanine oxidation and net phenylalanine balance over the range of intakes shown here with breakpoints at 1·46 and 1·53 g/ kg/d, respectively^(112)^. (d) The protein intake required to maximise whole-body anabolism during post-exercise recovery in resistance-trained men with high habitual intakes, with breakpoint analysis of the *F* ^13^CO_2_ in breath and net phenylalanine balance over the range of intakes shown here and with the breakpoints at an intake of 2·0 g/kg/d^(116)^. (e) The extent of any attenuation of post-exercise anabolism in resistance-trained men after an acute reduction in habitual protein intake^(117)^. All subjects were studied after a controlled intake for 2 d of a high-protein diet at 2·2 g protein/kg/d assumed to be equivalent to their habitual intake. They then undertook metabolic trials with small meals at a single intake of the amino acid mixture (with the usual fixed intake of phenylalanine and tyrosine) in a cross-over design. The high-protein phase involved an intake equivalent to 2·2 g/kg/d (H). The moderate phase involved an intake equivalent to 1·2 g/kg/d fed over a 5-d period with IAAO studies on days 1, 3 and 5 (M1, M3 and M5). Phenylalanine oxidation and net balance were measured at these single intakes on each occasion. Oxidation was higher with M1 compared with H but fell at M3 and M5 so that net phenylalanine balance improved on the moderate intake with time, with M5 not different from H. The authors argued that this demonstrated that adaptation from the high to the moderate protein intake was occurring over the 5 d. No explanation is given about why these studies of post-exercise anabolism at a single protein intake were carried out with the phenylalanine and tyrosine-limited amino acid mixture rather than with a complete amino acid mixture.

Further insight into this application of IAAO can be obtained from its application to various groups of athletes. After earlier studies of post-exercise anabolism in children^(107,108)^, Daniel Moore’s group adopted the minimally invasive IAAO approach with fixed intakes of phenylalanine (30·5 mg/kg/d) and tyrosine (40 mg/kg/d) and varying intakes of the amino acid mixture to investigate protein requirements in endurance athletes^(113)^ and female athletes^(115)^, and post-exercise anabolism in various groups: adults and adolescents^(111)^, resistance-trained females^(112)^, resistance-trained men with high habitual protein intakes^(116)^ and in the same group after a reduction in habitual intake^(117)^. However, in these studies, the focus was on post-exercise NPU (phenylalanine intake-phenylalanine oxidation) with the ‘net protein balance’ examined by breakpoint analysis over a range of the amino acid mixture intakes, between 0·2 and 2·8 g/kg/d. As shown in Fig. 11, this design, exactly the same as that shown in Fig. 9 involving untrained men, but with a higher range of intakes, would have resulted in the same variable phenylalanine deficiency at all intakes ≥ 0·9 g/kg/d becoming severe at the highest intake. Nevertheless, breakpoints in phenylalanine oxidation and net phenylalanine balance were identified at 1·43 g/kg/d^(115)^, 1·53 g/kg/d^(101,102)^ and 2·0 g/kg/d^(116)^, higher breakpoints compared with untrained men shown in Fig. 9. A potential explanation for this was indicated by a further study from the group. This was a single-intake study in resistance-trained men habituated to a high-protein intake^(117)^. They were fed the standard IAAO regime of repeated small meals at their habitual intake level (2·2 g/kg/d) and exhibited low rates of phenylalanine oxidation commensurate with 80 % of the phenylalanine intake utilised. However when switched to a lower intake of 1·2 g/kg/d and tested on days 1, 3 and 5 with this lower intake, they exhibited a near 100 % increase in oxidation and a marked reduction in net balance on day 1 but with adaptive reductions in oxidation at day 3 and 5 to levels of oxidation and net balance which were only slightly (not significantly) different to the high-protein responses. Because at each of these intake levels, 1·2 and 2·2 g protein/kg/d, the fixed 30·5 mg phenylalanine would have been limiting for complete utilisation of the protein intakes (as shown in Fig. 11), a similar response in terms of oxidation and net phenylalanine balance would have been expected. The authors concluded that post-exercise whole-body anabolism was attenuated following a reduction in protein intake in resistance-trained men and may require about 3–5 d to adapt. In fact, it could be argued that complete adaptation would have required > 5 d. They suggested that their observations of apparent protein requirements of athletes after exercise^(110,112,113,115)^, which were higher than those in untrained subjects^(10)^, could have reflected a lack of adaptation to the lower intake levels of the IAAO protocol.

**Fig. 12.**
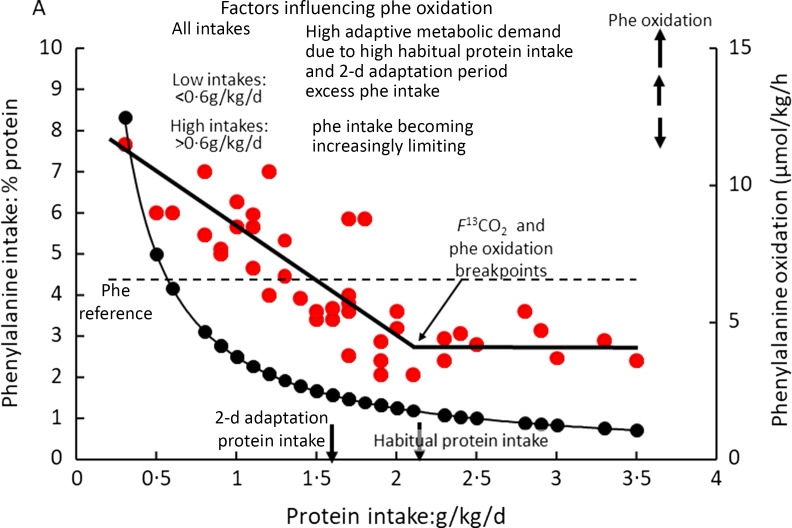
Factors influencing phenylalanine oxidation in IAAO studies on athletes^(114)^. Design and phenylalanine oxidation data from IAAO studies of the protein requirement of endurance-trained men with high habitual protein intakes. Phenylalanine intake is shown as a % of the amino acid mixture 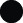, and phenylalanine oxidation is shown as µmol/kg/h 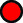. The design included a lower fixed intake of phenylalanine, 25 mg/kg/d compared with 30·5 mg/kg/d in most previous studies, so that as shown, the amino acid mixture became limiting for protein deposition at a lower intake (> 0·6 g/kg/d). Nevertheless, the breakpoints in both the phenylalanine oxidation and the *F* ^13^CO_2_ excretion in breath(not shown) (2·1 g/kg/d) in each case were at more than twice the intakes shown in Fig. 9. This was because phenylalanine oxidation did not fall as rapidly, remaining high in some subjects with intakes in which phenylalanine was grossly deficient for protein deposition. Potential explanations are shown in terms of the factors which influence phenylalanine oxidation in these studies. At all intakes, a high adaptive metabolic demand due to their high habitual protein intake (2·1 g/kg/d) would increase oxidation. The 2 d at 1·6 g/kg/d would not have been expected to lower this much during the feeding trials. At low intakes, < 0·6 g/kg/d, phenylalanine intakes would have been in excess compared with the composition of protein deposited, with oxidation its only fate so this would increase oxidation. At higher intakes, > 0·6 g/kg/d (the majority of subjects), phenylalanine intakes would have become increasingly deficient limiting net protein deposition with concentrations likely to fall, and this would lower oxidation. Values for phenylalanine oxidation redrawn from Bandegan et al. 2019^(114)^.

This latter study of Moore’s group^(117)^ is pertinent to two studies of the protein requirements of bodybuilders^(92)^, and endurance athletes^(114)^, also subjects with a habitually high-protein intake. The study design in terms of the range of intakes fed were similar for each study, and the phenylalanine oxidation data for the second study^(114)^ is shown in Fig. 12. For reasons not explained, a lower fixed intake of phenylalanine was used in each of these studies, 25 mg/kg/d compared with the 30·5 mg/kg/d used in all previous studies. This meant that in theory, as shown in Fig. 12, phenylalanine became limiting for protein deposition at a lower intake, > 0·6 g/kg/d compared with > 0·9 g/kg/d shown in Figs. 9 and 11. However, this did not lower the breakpoint in phenylalanine oxidation: on the contrary, it was much higher than in the normal men and comparable to the studies on athletes shown in Fig. 11. Examination of the phenylalanine oxidation in Fig. 12 shows it remained high, although falling, at amino acid intakes at which the phenylalanine intake was obviously deficient, that is, markedly below the reference phenylalanine level (the content in tissue protein). The breakpoints in oxidation at which a low constant level was reached, 2·1 g/kg/d, were intakes of the amino acid mixture in which phenylalanine, at 25 mg/kg/d, was unarguably deficient for protein deposition, allowing a maximum of 0·57 g tissue protein deposition if no oxidation occurred. Indeed given that there was some continuing phenylalanine oxidation, which can be shown to be equivalent to 33 % of the phenylalanine intake, actual net balance was equivalent to protein deposition of only 0·38 g/kg/d (assuming the oxidation rates are accurate).

In this and the preceding similar study^(92)^, the subjects had high habitual protein intakes of 2·1 g/kg/d and a 2-d adaptation period prior to the IAAO studies at intakes of 1·5 g/kg/d for the bodybuilders^(103)^, and 1·6 g/kg/d for the endurance athletes^(114)^, shown in Fig. 12. The factors likely to influence phenylalanine oxidation in these studies are shown in Fig. 12. Increased oxidation would have been likely at all intakes because of a lack of adaptation from a high adaptive MD as demonstrated by Tinline-Goodfellow et al.^(117)^. Increased oxidation would also have occurred because of the excess phenylalanine intake at low-protein intakes < 0·6 g/kg/d but would have been expected to fall at all higher intakes as it became limiting for net protein synthesis. Thus, these three factors acting together would have influenced the shapes of the phenylalanine oxidation plot shown in Fig. 12.

In a recent review of whether lengthy periods of adaptation are needed prior to IAAO studies of EAA requirements, the authors discuss a wide range of animal and human studies which have considered the need for prior adaptation as background to their argument for the need for only 2 d adaptation at 1·0 g/kg/d in such studies^(23)^, although they make little reference to IAAO studies of the protein requirement. They do cite the study discussed above by Tinline-Goodfellow et al^(117)^ as support for a 2-d period but do not address its findings that 5 d or more may be necessary for subjects on habitually high-protein diets; nor do they refer to their own adoption of a higher intake of 1·5 g/kg/d for 2 d for subjects on habitually high-protein intakes in the study shown in Fig. 12. The documentation, several decades ago, of a sustained fall in both post-absorptive and post-prandial leucine oxidation over 14 d after a switch from 1·8 to 0·8 g protein/kg/d^(41)^ is dismissed as ‘challenging’, and they argue that because of weight loss of the subjects ‘such data cannot be used to resolve the issue of the time required to achieve steady-state leucine oxidation or N-excretion to determine the EAA requirement in healthy humans’. However, they do not comment on the other findings in these studies that diurnal changes in oxidation and nitrogen losses of increasing amplitude with increasing protein intakes were reported in subjects fed for 12 d a wide range of protein intakes who maintained weight and FFM on each diet^(39,40)^, showing that the weight loss was a transient response during the adaptation.

### Conclusions on human indicator amino acid oxidation studies of protein requirements

**Fig. 13.**
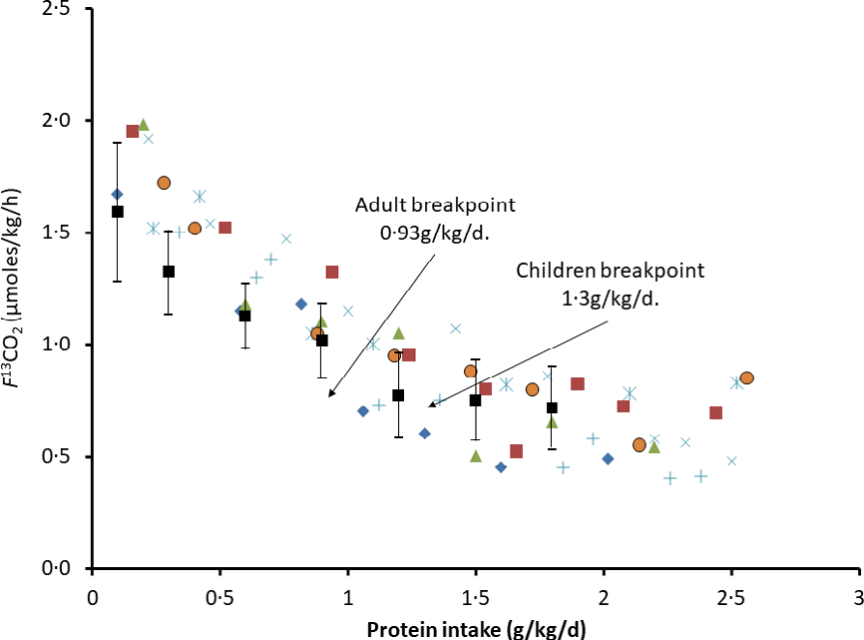
Results of IAAO studies of protein requirements of children and adult men^(10,118)^. Two studies with similar designs, showing phenylalanine oxidation (as *f* ^13^CO_2_) as a function of the intake of the amino acid mixture based on egg protein containing a fixed amount of phenylalanine, at 30·5 mg/kg/d in each case and tyrosine at 40 mg/kg/d in the adults and 61 mg/kg/d in the children. Both the school children (shown as individual results redrawn from Elango et al. 2011^(118)^ and the adult males shown as mean values ±1 sd (*n* 8 per mean) redrawn from Humayun et al. 2007^(10)^) were fed repeated small meals containing increasing intakes of the amino acid mixture On the basis of tissue protein phenylalanine content shown in Table 2 (44 mg/g), phenylalanine would become limiting at intakes > 0·7 g/kg/d, hence the low and constant oxidation values at intakes ≥ 1 g/kg/d at a rate which appears similar for the two group. However, the biphasic regression analysis showed a higher breakpoint for the children than for the adults. Background protein intakes were reported at 1·5 g/kg/d for the children, and they were fed such intakes for 2 d before the studies whilst the adults were fed a maintenance diet at 1 g/kg/d prior to the study. Oxidation rates at low-protein intakes appear to be somewhat higher for the children suggesting a higher adaptive metabolic demand as with the body builders shown in Fig. 12. Also as discussed in the text, the higher range of protein intakes in the children might also have increased the breakpoint value for the children.

The results presented in this section show that the application of IAAO to the determination of protein requirements differ in principle from its application to determination of EAA requirements discussed above. The response in terms of a breakpoint in indicator oxidation with this methodology has been achieved through feeding a constant phenylalanine intake with each increasing total amino acid intake which becomes insufficient to allow the efficient utilisation of the amino acid intake, and this reduced oxidation to a low constant level. This is clearly demonstrated in both the piglet studies in Fig. 8 and in the human studies in Figs. 9–12 with the exception of the study by Mao et al 2020^(66)^ which fed unlimited phenylalanine (Fig. 10). The fact that different breakpoints have been obtained in studies with different population groups fed the same limiting phenylalanine intake, as with the athletes illustrated in Figs. 11 and 12 does appear to reflect the influence of their high-protein background diets on their increased adaptive MD as was clearly demonstrated in one of Daniel Moore’s studies^(117)^. This would maintain higher oxidation rates at pre-breakpoint intakes shifting the breakpoint to the right (i.e. to higher intakes). Similarly whilst studies on healthy school children^(118)^ involved the same phenylalanine intake of 30·5 mg/kg/d as with the healthy adult men shown in Fig. 9 ^(10)^, different breakpoints in the *f* ^13^CO_2_data were identified: 1·3 g/kg/d in the children c.f. 0·93 g/kg/d in adults. This is shown in Fig. 13, in which it does appear that oxidation rates at low sub-breakpoint protein intakes are somewhat higher for the children suggesting a higher adaptive MD than in the adult men as with the body builders shown in Fig. 12. Background protein intakes were reported at 1·5 g/kg/d for the children, and they were fed such intakes for 2 d before the studies whilst the adults were fed a maintenance diet at 1 g/kg/d prior to the study. Although the phenylalanine flux and oxidation rates in the two studies were different (values for the children were half those in the adults, which would not be expected, and which was not explained), this would not be expected to influence the *f* ^13^CO_2_ values shown in Fig. 13. It was the case that the overall range of the amino acid intakes differed with higher intakes fed to the children, up to 2·6 g/kg/d, compared with 1·8 g in the adults, and given the way in which the biphasic regression analysis works in practice (as discussed in relation to Fig. 7), a greater range of intakes above the breakpoint could shift the breakpoint to the right. In any case, it is highly unlikely that there is a biological explanation of the differences in the breakpoints which relates to the protein requirements.

At the outset of this section which examined what might be expected to be observed during post-prandial studies of protein utilisation and amino acid oxidation, it was concluded that a response curve relating amino acid oxidation to protein intake was unlikely to reveal information about the protein requirement other than possibly the changes in the utilisation of meal protein as intakes increase. Clearly with the single exception of the study shown in Fig. 10 which fed unlimited amounts of phenylalanine, all IAAO studies of protein requirements have generated response curves with an undeniable breakpoint. However as shown here, it is indisputable that protein deposition will be limited at the higher intakes of the amino acid mixture because they would not contain sufficient phenylalanine to allow more than a limited amount of tissue protein deposition as demonstrated most obviously in Fig. 12. It is notable that in the only study where the indicator was not limiting^(66)^, there was no obvious breakpoint in the oxidation data (see Fig. 10). Because of this fundamental flaw in the design concept it is difficult to identify any value of these studies in defining protein requirements.

### Conclusions

At the outset, this review considered the questions what do we know about the impact of varying protein intakes on homoeostatic regulation of the FFM and what can post-prandial studies of amino acid and protein utilisation tell us about amino acid and protein requirements?

As to the first of these questions progress has been slow to develop any generally agreed framework for FFM homoeostasis since the concept of the protein-stat was introduced in 1995^(43)^. As recently discussed elsewhere^(44)^, the characteristics of child growth in relation to varying protein intakes in infancy and the development of excessive adiposity of which the low-protein intakes with breast-feeding protects against, identified by Koletzko as the Early Protein Hypothesis ^(119)^, are consistent with the central concept of the protein-stat that muscle growth is limited by bone length growth and protein intakes in excess of the MD can increase adiposity. Certainly, the changes observed in relative growth in length and muscle mass in puberty support the protein-stat concept. Thus, the existent of an upper limit to post-prandial protein deposition in both children and adults can be considered firmly established by the phenomenology of both childhood growth to adulthood and studies of healthy adult populations and their dietary protein intakes discussed above. Clearly although information is emerging on the mechanisms involved in such limitation of post-prandial protein deposition from both animal and human studies especially of MPS^(42,44,120)^, much remains to be revealed. The studies of leucine oxidation in response to feeding increasing protein intakes^(105,106)^ suggest that utilisation was falling at the higher intakes fed in these subjects.

As to post-prandial studies in relation to amino acid and protein requirements, the work described here needs to be viewed in the context of previous work. Following the 1973 FAO/WHO report on the protein requirement, which controversially derived a factorial protein requirement which was lower than previous values, there was an international effort to organise NB studies with a standardised protocol. These studies contributed to the revised higher adult value in the 1985 report^(121)^ which was similar to the current 2007 values^(1)^. This latter report derived its recommendations from the median value indicated by a meta-analysis of all suitable NB studies in healthy adults. Since then notwithstanding widespread criticisms of NB, no universally accepted alternative method for the protein requirement has emerged. After stable isotope tracer studies of amino acid and protein metabolism became widespread in the 1970–1980s, there is a large literature of exploratory investigations which to some extent culminated in the 24-h [^13^C-1] leucine balance studies of amino acid requirements mounted by Vernon Young and Anura Kurpad^(122)^, and these contributed to the derivation of values listed in the 2007 WHO report, as did some of the IAAO studies of the amino acid requirement^(1)^. However, no generally accepted alternative study to NB has emerged, especially in the context considered here, post-prandial studies of protein metabolism. As described in relation to Fig. 1, our own studies of PPU in the fed and fasted state^(52)^ did result in an experimental protocol allowing the assessment of apparent protein requirements (the requirement observed in subjects at their habitual protein intake) and how they changed with age^(59)^ and of the influence of protein quality on PPU^(51)^, results which allowed the elaboration of the adaptive MD model of the protein requirement^(53)^. Furthermore in the recent technical report from FAO^(67)^, a modified protocol was described to asses dietary protein quality. However, this overall body of work has had only a moderate impact, as indicated by citations of the work, and the methodology has not been adopted by others. It is to be hoped that this will change given the unresolved nature of many of the issues relating to amino acid and protein requirements.

As to IAAO studies, of which there is now a considerable body of work relating to the determination of requirements for both EAA and protein, this presentation has focused on an examination of the key principles as indicated by representative examples. From the outset, they represented a change from earlier isotope kinetic post-prandial studies in which the focus was on quantitative isotopic kinetic studies of responses to feeding, in terms of the fate of dietary protein intake and measurements of protein synthesis as discussed in the section on post-prandial metabolism above or in the 24 h [^13^C-1] leucine balance studies of Kurpad and Young^(94,98,123)^. Most IAAO studies have been qualitative, aimed at identifying a ‘response’, a change or breakpoint in oxidation of the indicator, with the rationale for the experimental design especially in relation to the amounts of the indicator fed, usually based on prior IAAO studies. Thus, calculations seldom appear to have been made about the implications of the breakpoint for net protein deposition and whether intakes of the indicator phenylalanine was itself limiting and an influence on the breakpoint. As shown here, simple calculations based on the likely EAA content of tissue protein deposited in these studies unequivocally show how the outcome of IAAO studies of requirements for both EAA and protein is influenced by the limiting intakes of the indicator. It is extremely difficult to understand the purpose of a protocol examining IAAO responses to intakes of amino acid mixtures up to equivalent protein intakes of 3·5 g/kg/d as in Fig. 12 with the phenylalanine intake fixed at 25 mg/kg/d only allowing a small fraction of the intake to be utilised. It is interesting to note that at least two groups adopting IAAO have decided not to limit phenylalanine intakes in their studies of lysine requirement^(86)^, or protein requirements^(66)^, although each adopted breakpoint analysis and obtained a breakpoint. Taken together the work summarised here has shown that whilst Bayley’s original piglet studies of EAA requirements to ensure maximum growth did result in a valid IAAO method and important results as far as their growth requirements, it is difficult to demonstrate a satisfactory outcome for most of the human applications of that approach, or for IAAO studies of the protein requirements either in the piglet or in human studies.

Clearly progress in this area, which is most obviously needed, requires a more forensic approach to past studies combined with new thinking.
